# From 1D Coordination Polymers to Metal Organic Frameworks by the Use of 2-Pyridyl Oximes

**DOI:** 10.3390/ma13184084

**Published:** 2020-09-14

**Authors:** Ioannis Mylonas-Margaritis, Auban Gérard, Katerina Skordi, Julia Mayans, Anastasios Tasiopoulos, Patrick McArdle, Constantina Papatriantafyllopoulou

**Affiliations:** 1 School of Chemistry, College of Science and Engineering, National University of Ireland Galway, SSPC, Synthesis and Solid State Pharmaceutical Centre, University Road, Galway H91 TK33, Ireland; i.mylonas-margaritis1@nuigalway.ie (I.M.-M.); auban.gerard@uha.fr (A.G.); patrick.mcardle@nuigalway.ie (P.M.); 2 Department of Chemistry, University of Cyprus, 1678 Nicosia, Cyprus; skordi.katerina@ucy.ac.cy (K.S.); atasio@ucy.ac.cy (A.T.); 3 Instituto de Ciencia Molecular (ICMol), Universidad de Valencia, Catedrático José Beltran 2, 46980 Paterna (Valencia), Spain; julia.mayans@qi.ub.edu

**Keywords:** coordination polymers, metal–organic frameworks (MOFs), carboxylates, pyridyl oximes, mixed-ligand, detection, encapsulation, iron(III), magnetism

## Abstract

The synthesis and characterization of coordination polymers and metal–organic frameworks (MOFs) has attracted a significant interest over the last decades due to their fascinating physical properties, as well as their use in a wide range of technological, environmental, and biomedical applications. The initial use of 2-pyridyl oximic ligands such as pyridine-2 amidoxime (H_2_pyaox) and 2-methyl pyridyl ketoxime (Hmpko) in combination with 1,2,4,5-benzene tetracarboxylic acid (pyromellitic acid), H_4_pma, provided access to nine new compounds whose structures and properties are discussed in detail. Among them, [Zn_2_(pma)(H_2_pyaox)_2_(H_2_O)_2_]_n_ (**3**) and [Cu_4_(OH)_2_(pma)(mpko)_2_]_n_ (**9**) are the first MOFs based on a 2-pyridyl oxime with **9** possessing a novel 3,4,5,8-c net topology. [Zn_2_(pma)(H_2_pyaox)_2_]_n_ (**2**), [Cu_2_(pma)(H_2_pyaox)_2_(DMF)_2_]_n_ (**6**), and [Cu_2_(pma)(Hmpko)_2_(DMF)_2_]_n_ (**8**) join a small family of coordination polymers containing an oximic ligand. **9** exhibits selectivity for Fe^III^ ions adsorption, as was demonstrated by a variety of techniques including UV-vis, EDX, and magnetism. DC magnetic susceptibility studies in **9** revealed the presence of strong antiferromagnetic interactions between the metal centers, which lead to a diamagnetic ground state; it was also found that the magnetic properties of **9** are affected by the amount of the encapsulated Fe^3+^ ions, which is a very desirable property for the development of magnetism-based sensors.

## 1. Introduction

The synthesis and characterization of metal coordination polymers has attracted an intense research interest over the recent years due to their applications in a variety of fields, including catalysis, drug delivery sensing, etc. [1,2,3,4,5,6,7]. The structure of such species is based on mononuclear or low nuclearity inorganic units that are held together through organic ligands forming multidimensional networks whose properties are strongly affected by the nature of the metal ions and the organic linkers. For example, 1D coordination polymers of paramagnetic metal ions can display single chain magnetism (SCM) behavior, i.e., they can exhibit slow relaxation of magnetization stemming from strong intrachain exchange interactions between high spin structural building units along the chain [8,9,10,11,12,13,14]. SCMs are excellent candidates for applications in high-density information storage, molecular spintronics, quantum computation, etc. [15,16,17,18,19,20,21]. As the dimensionality of the network increases, the induced porosity can be combined with magnetism and/or another physical property (e.g., photoluminescence, etc.), leading to the development of hybrid multifunctional materials. The synergistic effect between two different properties often enhances the performance of such species in a variety of significant applications including sensing, catalysis, drug delivery, spintronics, photonics, and others.

One growing family of multifunctional materials is the one of metal–organic frameworks (MOFs) [22,23,24]; MOFs are crystalline porous materials built from inorganic secondary building units (SBUs) that are connected through polytopic organic linkers. They display a range of appealing structural features such as large surface area, high porosity, flexible structure, an amphiphilic internal microenvironment, and the possibility of introducing functional groups in the pores and frameworks in a spatially controlled way. The unique properties of MOFs and their structural tuneability [25,26,27,28,29] make these materials especially suitable for encapsulating a large variety of guest molecules, and hence they are promising candidates for a plethora of environmental and biomedical applications [30,31,32,33,34,35]. Restricting further discussion to the ability of MOFs to capture and remove toxic compounds from the environment, MOFs display all the desirable features in terms of water stability, porosity, and surface area, and can be used as alternate adsorbents for the adsorption and removal of toxic species [36,37,38,39,40,41,42,43,44,45,46,47]. In addition to their capturing capacity, MOFs often have sensing properties, which are based on the change to the physical properties caused by the encapsulated metal ion [48,49]. Although there are a few reports that take advantage of the impact of the guest species on the color, electrochemical, and other properties for the development of sensors, the vast majority of them display luminescence-based sensing, which happens due to the change of the emission and/or lifetime after the toxic chemical capture. One recent such example is the FJI-C8 MOF, which exhibits a high sensitivity for Fe^3+^ with the detection limit being 0.0233 mM [48]; it contains a π-conjugated aromatic ligand with free/uncoordinated N- and O-atoms, which interact strongly with the encapsulated Fe^3+^ ions affecting the emission spectrum of the anionic FJI-C8 MOF. Other luminescent-active MOFs have also been studied as metal ion sensors, including examples of Ru-, Ln-MOFs, etc. [50,51,52,53,54].

The wide range of applications of MOFs and coordination polymers constitute an increasing need for the development of new such species with suitable porosity, high stability, and fine-tuning properties [55,56,57,58,59,60,61,62,63]. A large number of organic linkers have now been employed in MOFs synthesis including imidazolates, pyridine, carboxylates, etc. [64,65,66,67,68,69,70,71,72,73,74], with the latter being one of the most commonly used, resulting in MOFs with a wide range of pores sizes and shapes. The decoration of the ligands with free (non-coordinated) groups provide valuable opportunity for the insertion of additional functionalities to the framework, which can be useful for environmental and biomedical applications; for example, the presence of pendent sulfonates enhances the MOF ability for the adsorption and removal of heavy metals from aqueous systems, whereas the presence of π-electron-rich N-donor groups enhances the sensing performance towards electron-deficient nitroaromatic explosives [75,76].

Although the impact of the ligand combination on the framework topology and porosity has been well investigated, this is not the case for the nuclearity and properties of the SBU itself [72,73,74,75,76,77,78]. In fact, the SBUs significantly affect the properties of the overall framework; it has been shown that the presence of an heterometal has a positive impact on the breathing effect, whereas the increase of the metal nuclearity is possible to enhance the MOF’s pore size and surface area [79,80,81]. Thus, it is essential that ligands with high bridging capability that have the potential to lead to species with unprecedented metal topologies and/or high nuclearity SBUs be introduced into this field; to this purpose, we decided to use 2-pyridyl oximes for the isolation and characterization of new MOFs and coordination polymers. 2-pyridyl oximes is a family of ligands that have been extensively used in metal cluster chemistry due to their ability to bridge a large number of metal ions and, often, promote ferromagnetic interactions between the metal centers [82,83,84,85,86,87,88,89,90,91,92,93,94,95]. Although the use of such ligands has led to significant breakthroughs in the areas of single-molecule and single-chain magnetism, they have never been employed for MOF synthesis [82,83,84,85,86,87,88,89,90,91,92,93,94,95].

**Scheme 1:**
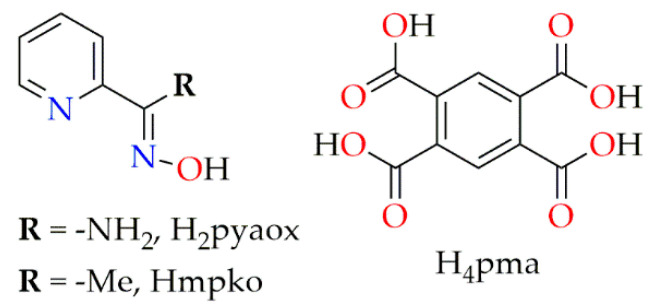
Schematic representation of the 2-pyridyl oximes (**left**) and the ligand H_4_pma (**right**) discussed in this work.

Herein, we report on the synthesis, structural characterization, and physical properties of nine new coordination polymers, including the first oxime-based MOFs, by the use of a 2-pyridyl oxime (pyridine-2 amidoxime, H_2_pyaox, and 2-methyl pyridyl ketoxime, Hmpko) in combination with 1,2,4,5-benzene tetracarboxylic acid (pyromellitic acid), H_4_pma (Scheme 1); the latter has been employed in the field of MOFs [96,97,98,99,100,101,102,103,104], but its combination with an oximic ligand is unexplored. The 3D MOF [Cu_4_(OH)_2_(pma)(mpko)_2_]_n_ (**9**) displays selectivity for Fe^3+^ adsorption.

## 2. Materials and Methods 

### 2.1. Materials, Physical, and Spectroscopic Measurements

All the manipulations were performed under aerobic conditions using materials (reagent grade) and solvents as received. Hmpko and H_2_pyaox was prepared as described elsewhere [105,106]. WARNING: Perchlorate salts are potentially explosive; such compounds should be used in small quantities and treated with utmost care at all times.

Elemental analysis (C, H, N) were performed by the in-house facilities of National University of Ireland Galway, School of Chemistry. IR spectra (4000–400 cm^−1^) were recorded on a PerkinElmer Spectrum 400 FT-IR spectrometer. Powder X-ray diffraction data (pxrd) were collected using an Inex Equinoz 6000 diffractometer. Solid-state, variable-temperature, and variable-field magnetic data were collected on powdered samples using an MPMS5 Quantum Design magnetometer operating at 0.03 T in the 300 to 2.0 K range. Diamagnetic corrections were applied to the observed susceptibilities using Pascal’s constants. TGA experiments were performed on a STA625 thermal analyzer from Rheometric Scientific (Piscataway, New Jersey). The heating rate was kept constant at 10 °C/min, and all runs were carried out between 20–600 °C. The measurements were made in open aluminum crucibles, nitrogen was purged in ambient mode, and calibration was performed using an indium standard.

### 2.2. Compound Synthesis

#### 2.2.1. Synthesis of [Zn(H_2_pma)(H_2_pyaox)(H_2_O)_2_] (**1**)

Zn(ClO_4_)_2_·6H_2_O (0.149 g, 0.4 mmol) and H_2_pyaox (0.027 g, 0.20 mmol) were dissolved in H_2_O (20 mL). The resultant solution was put in the oven and heated at 100 °C for 1 h. Then, solid H_4_pma (0.025 g, 0.1 mmol) was added, the solution was stirred for 15 min, and the vial was left at R.T. for 24 h, after which X-ray quality colorless crystal needles of **1** were formed. The crystals were collected by filtration, washed with cold MeCN (2 mL) and Et_2_O (2 × 5 mL), and dried in air. Yield 80%. Anal. Calc. for **1**: C, 39.16; H, 3.08; N, 8.56. Found: C, 39.47; H, 3.29; N, 8.17%. IR data: *v* (cm^−1^) = 3504m, 3403m, 3123w, 1725s, 1669m, 1609m, 1583s, 1497s, 1414m, 1376s, 1321m, 1295w, 1267w, 1218s, 1168s, 1118s, 1087m, 1062w, 1029s, 1015m, 944m, 916m, 858b, 816w, 794s, 761m, 744m, 725w, 701w, 679w, 667w, 651w.

#### 2.2.2. Synthesis of [Zn_2_(pma)(H_2_pyaox)_2_]_n_ (**2**)

Zn(ClO_4_)_2_·6H_2_O (0.149 g, 0.4 mmol) and H_2_pyaox (0.027 g, 0.20 mmol) were dissolved in H_2_O (10 mL). The resultant solution was put in the oven and heated at 100 °C for 1 h. Solid H_4_pma (0.025 g, 0.1 mmol) was then added and the vial was placed into the oven for 24 h, after which X-ray quality colorless crystal needles of **2** were formed. The crystals were collected by filtration, washed with cold MeCN (2 mL) and Et_2_O (2 × 5 mL), and dried in air. Yield 39%. Anal. Calc. for **2**: C, 40.33; H, 2.46; N, 12.83. Found: C, 40.56; H, 2.49; N, 13.09%. IR data: *v* (cm^−1^) = 3399w, 3215w, 2931w, 1603s, 1402m, 1369s, 1322w, 1253w, 1138w, 1103m, 1061w, 1039m, 915w, 865m, 813m, 783w, 762w, 739w, 683m, 664w.

#### 2.2.3. Synthesis of [Zn_2_(pma)(H_2_pyaox)_2_(H_2_O)_2_]_n_ (**3**)

Zn(ClO_4_)_2_·6H_2_O (0.149 g, 0.4 mmol) and H_2_pyaox (0.027 g, 0.20 mmol) were dissolved in H_2_O (20 mL). The resultant solution was put in the oven and heated at 100 °C for 1 h. Solid H_4_pma (0.025 g, 0.1 mmol) was then added and the vial was placed into the oven for 2 h, after which X-ray quality colorless polyhedral crystals of **3** were formed. The crystals were collected by filtration, washed with cold MeCN (2 mL) and Et_2_O (2 × 5 mL), and dried in air. Yield 50%. Anal. Calc. for **3**: C, 38.23; H, 2.92; N, 12.16. Found: C, 37.91; H, 2.63; N, 11.93%. IR data: *v* (cm^−1^) = 3423w, 3316w, 3202w, 2786w, 1675m, 1648w, 1619w, 1575w, 1548s, 1494s, 1415w, 1372s, 1324m, 1292w, 1267w, 1178w, 1158w, 1138m, 1097w, 1040s, 980w, 942m, 874m, 850w, 813m, 786s, 747s, 690m, 651w.

#### 2.2.4. Synthesis of [Co_2_(pma)(H_2_pyaox)_2_(H_2_O)_6_] (**4**)

Co(ClO_4_)_2_·6H_2_O (0.146 g, 0.4 mmol) and H_2_pyaox (0.027 g, 0.2 mol) were dissolved in DMF/H_2_O (10/10 mL). The resultant solution was put in the oven and heated at 100 °C for 1 h. Solid H_4_pma (0.025 g, 0.1 mmol) was then added and the vial was placed into the oven for 24 h, after which X-ray quality orange crystals of **4** were formed. The crystals were collected by filtration, washed with cold MeCN (2 mL) and Et_2_O (2 × 5 mL), and dried in air. Yield 45%. Anal. Calc. for **4**: C, 35.22; H, 3.76; N, 11.20. Found: C, 35.17; H, 3.68; N, 11.19%. IR data: *v* (cm^−1^) = 3421w, 3308w, 3202w, 2780w, 1667w, 1648w, 1618w, 1573w, 1552s, 1493m, 1412m, 1368s, 1324m, 1293w, 1157w, 1137m, 1096m, 1096w, 1034s, 981m, 942m, 872m, 829w, 812m, 784s, 748m, 689m, 665w.

#### 2.2.5. Synthesis of [Mn_2_(pma)(H_2_pyaox)_2_(H_2_O)_6_] (**5**)

Mn(ClO_4_)_2_·6H_2_O (0.102 g, 0.4 mmol) and H_2_pyaox (0.0549 g, 0.4 mmol) were dissolved in H_2_O (10 mL). The resultant yellow solution was put in the oven and heated at 100 °C for 1 h. Then, solid H_4_pma (0.025 g, 0.1 mmol) was added and the vial was placed into the oven for 24 h, after which X-ray quality colorless needles of **5** were formed. The crystals were collected by filtration, washed with cold MeCN (2 mL) and Et_2_O (2 × 5 mL), and dried in air. Yield 50%. Anal. Cal. for **5**: C, 35.59; H, 3.80; N, 11.32. Found: C, 35.72; H, 4,06; N, 11.23%. IR data: *v* (cm^−1^) = 3358w, 3280w, 2814w, 2229w, 1859m, 1667m, 1649m, 1602m, 1571w, 1545s, 1476s, 1440m, 1418s, 1368s, 1314s, 1259m, 1193w, 1175w, 1165m, 1141s, 1101m, 1071w, 1042s, 968w, 923m, 896w, 853m, 837w, 811w, 792m, 774s, 745s, 689s, 669m, 660w, 651w.

#### 2.2.6. Synthesis of [Cu_2_(pma)(H_2_pyaox)_2_(DMF)_2_]_n_ (**6**)

Cu(ClO_4_)_2_·6H_2_O (0.149 g, 0.4 mmol) and H_2_pyaox (0.027 g, 0.20 mmol) were dissolved in DMF/H_2_O (7/7 mL). The resultant solution was put in the oven and heated at 100 °C for 1 h. Then, solid H_4_pma (0.025 g, 0.1 mmol) was added and the vial was placed into the oven for 24 h, after which X-ray quality green crystal needless of **6** were formed. The crystals were collected by filtration, washed with cold MeCN (2 mL) and Et_2_O (2 × 5 mL), and dried in air. Yield 39%. Anal. Calc. for **6**: C, 42.16; H, 3.79; N, 14.05. Found: C, 41.87; H, 3.64; N, 13.83%. IR data: *v* (cm^−1^) = 3355w, 3226w, 1647s, 1605m, 1483w, 1437w, 1407w, 1363m, 1322w, 1300w, 1245m, 1211m, 1142w, 1098s, 1063w, 1041m, 945w, 928w, 893w, 860w, 844w, 811w, 784m.

#### 2.2.7. Synthesis of [Zn_2_(pma)(Hmpko)_2_(H_2_O)_4_]·2H_2_O (**7**∙2H_2_O) 

Zn(ClO_4_)_2_·6H_2_O (0.149 g, 0.4 mmol) and Hmpko (0.027 g, 0.20 mmol) were dissolved in DMF/H_2_O (7/7 mL). The resultant solution was put in the oven and heated at 100 °C for 1 h. Solid H_4_pma (0.025 g, 0.1 mmol) was then added and the vial was placed into the oven for 24 h, after which X-ray quality colorless crystal needles of **7** were formed. The crystals were collected by filtration, washed with cold MeCN (2 mL) and Et_2_O (2 × 5 mL), and dried in air. Yield 40%. Anal. Calc. for **7**: C, 37.87; H, 3.97; N, 7.36. Found: C, 38.10; H, 3.93; N, 7.91%. IR data: *v* (cm^−1^) = 3102w, 1661m, 1619w, 1548s, 1490m, 1428m, 1375s, 1326s, 1259w, 1187w, 1140m, 1102w, 1046m, 1024w, 969w, 919w, 871m, 827m, 814m, 780w, 762w, 745w, 689m, 664w, 651w.

#### 2.2.8. Synthesis of [Cu(pma)_0.5_(Hmpko)(DMF)]_n_ (**8**)

Cu(ClO_4_)_2_·6H_2_O (0.148 g, 0.4 mmol) was added to a solution of Hmpko (0.027 g, 0.2 mmol) in DMF/H_2_O (10/10 mL). The vial was placed into the oven (100 °C) and, after 1 h, H_4_pma (0.025 g, 0.1 mmol) was added. The vial was then left in the oven for a further 1 h, after which X-ray quality green crystals of **8** were observed. The crystals were collected by filtration, washed with cold MeCN (2 mL) and Et_2_O (2 × 5 mL), and dried in air. Yield 45%. Anal. Calc. for **8:** C, 45.28; H, 4.05; N, 10.56. Found: C, 45.41; H, 4.06; N, 10.64%. IR data: *v* (cm^−1^) = 3073w, 2342w, 2202w, 2168w, 2068w, 2018w, 1990w, 1623s, 1603w, 1572s, 1480m, 1438w, 1421w, 1399w, 1358bs, 1329w, 1308w, 1297w, 1273w, 1250w, 1170m, 1145m, 1101s, 1064s, 1047m, 1034w, 971w, 925s, 860s, 820w, 811s, 788s, 760s, 715m, 685s, 664s.

#### 2.2.9. Synthesis of [Cu_4_(OH)_2_(pma)(mpko)_2_]_n_ (**9**)

Method A: **9** was prepared in the same manner as **8**, but was left in the oven for 4 h, instead of 1 h in **9**. The crystals were collected by filtration, washed with cold MeCN (2 mL) and Et_2_O (2 × 5 mL), and dried in air. Yield 80%. Anal. Calc. for **9:** C, 35.65; H, 2.24; N, 6.93. Found: C, 35.73; H, 2.52; N, 6.33%. IR data: *v* (cm^−1^) = 3339b,1651w, 1616w, 1602w, 1567w, 1547w, 1481s, 1440m, 1416s, 1360s, 1316s, 1270w, 1165s, 1140m, 1102w, 1090m, 1046w, 1023w, 969b, 918m, 899w, 861m, 808s, 773s, 757m, 747m, 707s, 688m, 666w.

Method B: **9** (0.0760 g, ~0.1 mmol) in DMF/H_2_O (10/10 mL) was placed into the oven (100 °C) for 4 h, after which time green crystals of **9** were formed; the crystals were collected by filtration washed with MeCN (2 × 5 mL) and dried under vacuum. Yield: 80%. The product was characterized by PXRD and IR comparison with the authentic material.

### 2.3. Single-Crystal X-ray Crystallography

Single-crystal diffraction data for **1**, **5**, **6**, and **8** were collected in an Oxford Diffraction Xcalibur CCD diffractometer, whereas crystallographic data for **2–4**, **7**, and **9** were collected in an Oxford-Diffraction SuperNova A diffractometer. Mo Ka radiation (λ = 0.71073 Å) was used for **1**, **3**, **5**, **6**, and **8**, and Cu Ka radiation (λ = 1.54184 Å) was used for **2**, **4**, **7**, and **9**. The structures were solved using SHELXT [107], embedded in the OSCAIL software [108]. The non-H atoms were treated anisotropically, whereas the hydrogen atoms were placed in calculated, ideal positions and refined as riding on their respective carbon atoms. Molecular graphics were produced with DIAMOND [109]. 

Unit cell data and structure refinement details for **1–9** are listed in Table 1. CIF files can be obtained free of charge from the Cambridge Crystallographic Data Centre, Cambridge, UK with the REF codes 2022403-2022411 for **1**–**9**, respectively.

### 2.4. Metal Ion and 2-methyluracil Adsorption Kinetic and Thermodynamic Studies

The metal adsorption capacity of **9** was investigated as described below: a salt of a metal ion (0.027 g, 0.1 mmol for FeCl_3_; 0.040 g, 0.1 mmol for Fe(NO_3_)_3_) was added to a glass vial containing distilled H_2_O (10 mL) and stirred until all solid is dissolved. Solid **9** (0.242 g) was then added and the mixture was left stirring at room temperature. For the kinetic study, small volumes of aliquots were taken at designated time intervals, centrifuged, and the metal content in the supernatant solution was determined by spectroscopic (UV-vis) techniques. For the thermodynamic study, the same procedure was repeated with varying **9**: metal ratios; the mixture was stirred for 20 min, filtered, and the filtrate was analyzed for its metal content. The metal encapsulation was also confirmed by magnetism studies. 

The 2-methyluracil adsorption capacity of **9** was investigated following the same method used for the metal adsorption studies.

## 3. Results and Discussion

### 3.1. Synthetic Discussion

We have developed an intense interest over the last years in the synthesis of metal clusters and SMMs by the use of 2-pyridyl oximes as bridging ligands. These research efforts have resulted in a large number of new homo- and heterometallic new species with interesting structural features and magnetic properties, e.g., Ni_5_, Ni_12_, Ni_16_, Ni_2_Ln_2_, Ni_8_Ln_8_, Ni_2_Mn_2_, Mn_8_, etc. [83,84,85,86,87,88,89]. We recently decided to explore the ability of such ligands to favor the formation of MOFs, when combined with di- and tricarboxylic ligands, such as 1,4-benzenedicarboxylic and 1,3,5-benzenetricarboxylic acid [110]. This study yielded a new family of 1D chains and prompted us to expand this work using a tetracarboxylic ligand with higher bridging capability, in order to further increase the potential of the reaction system to provide access to new MOFs. The ligand that was chosen, and discussed herein, is the pyromellitic acid, H_4_pma. A variety of experiments were performed, studying how the different synthetic parameters (presence/absence or kind of base, molar ratio of the reactants, metal sources, etc.) affect the identity of the isolated product. In particular, the use of other metal sources instead of perchlorates (e.g., chlorides, nitrates, acetates, etc.) led to the precipitation of amorphous materials that could not be further characterized.

In order to ensure the coordination of both the oxime and carboxylic ligand in the metal center, the solution containing the metal salt and the oxime was left under stirring for at least one hour before the addition of the H_4_pma. It is noteworthy that the appropriate ratio of oxime/H_4_pma, which is able to yield binary species, was found to be 2:1; a 1:1 ratio results in known compounds that contain only the teracarboxylic ligand, whereas a high excess of oxime leads to the formation of oximato metal complexes, preventing the coordination of the ligand H_4_pma either in its neutral or anionic form.

The reaction mixture of Zn(ClO_4_)_2_·6H_2_O/H_2_pyaox/H_4_pma (4:2:1) in H_2_O gave a colorless solution from which crystals of [Zn(H_2_pma)(H_2_pyaox)(H_2_O)_2_] (**1**) were subsequently isolated. Following a similar reaction but by increasing the reaction temperature from R.T. to 100 °C, compounds [Zn_2_(pma)(H_2_pyaox)_2_]_n_ (**2**) and [Zn_2_(pma)(H_2_pyaox)_2_(H_2_O)_2_]_n_ (**3**) were isolated, depending on the reaction time; **2** is an 1D polymer and is formed after 24 h, whereas **3** is a 2D MOF, which is formed after 2 h of reaction. The stoichiometric equation of the reactions that lead to the formation of **1**–**3** is represented in Equations (1)–(3).

(1) $$Zn{\left({\mathrm{ClO}}_{4}\right)}_{2}\cdot 6H_{2}O\;+{\;H}_{2}\mathrm{pyaox}\;+{\;H}_{4}\mathrm{pma}\;\overset{\;H_{2}O\;}{\to }\;[\mathrm{Zn}\left(H_{2}\mathrm{pma}\right)\left(H_{2}\mathrm{pyaox}\right)\left(H_{2}O{)}_{2}\right]\;+\;4{\;H}_{2}O\;+\;2{\;H}^{+}\;+\;2{\;\mathrm{ClO}}_{4}^{-}\;1$$

(2) $$2\;\mathrm{Zn}{\left({\mathrm{ClO}}_{4}\right)}_{2}\cdot 6H_{2}O\;+\;2{\;H}_{2}\mathrm{pyaox}\;+{\;H}_{4}\mathrm{pma}\;\overset{\;H_{2}O\;}{\to }{\;[{\mathrm{Zn}}_{2}\left(\mathrm{pma}\right){\left(H_{2}\mathrm{pyaox}\right)}_{2}]}_{n}\;+\;12{\;H}_{2}O\;+\;4{\;H}^{+}\;+\;4{\;\mathrm{ClO}}_{4}^{-}\;2$$

(3) $$2\;\mathrm{Zn}{\left({\mathrm{ClO}}_{4}\right)}_{2}\cdot 6H_{2}O\;+\;2{\;H}_{2}\mathrm{pyaox}\;+{\;H}_{4}\mathrm{pma}\;\overset{\;H_{2}O\;}{\to }{\;[{\mathrm{Zn}}_{2}\left(\mathrm{pma}\right){\left(H_{2}\mathrm{pyaox}\right)}_{2}{\left(H_{2}O\right)}_{2}]}_{n}\;+\;10{\;H}_{2}O\;+\;4{\;H}^{+}\;+\;4{\;\mathrm{ClO}}_{4}^{-}\;3$$

After the determination of the crystal structures of **1**–**3**, which revealed that small modification in the reaction conditions affect the dimensionality of the compound (**1**, 0D; **2**, 1D; **3**, 2D), we decided to investigate the impact of the kind of the metal ion on the identity of the isolated products. Thus, compounds [Co_2_(pma)(H_2_pyaox)_2_(H_2_O)_6_] (**4**), [Mn_2_(pma)(H_2_pyaox)_2_(H_2_O)_6_] (**5**), and [Cu_2_(pma)(H_2_pyaox)_2_(DMF)_2_]_n_ (**6**) were isolated in good yield by a similar reaction to the one that provided access to **1** and **2** (Equations (4) and (5)). **4** and **5** are discrete complexes (0D), whereas **6** is a 1D double chain.

(4) $$2\;M{\left({\mathrm{ClO}}_{4}\right)}_{2}\cdot 6H_{2}O\;+\;2{\;H}_{2}\mathrm{pyaox}\;+{\;H}_{4}\mathrm{pma}\;\overset{\;H_{2}O/\mathrm{DMF}\;}{\to }\;[M_{2}\left(\mathrm{pma}\right){\left(H_{2}\mathrm{pyaox}\right)}_{2}\left(H_{2}O{)}_{6}\right]\;+\;6{\;H}_{2}O\;+\;4{\;H}^{+}\;+\;4{\;\mathrm{ClO}}_{4}^{-}\;M\;={\;\mathrm{Co}}^{\mathrm{II}},\;4;{\;\mathrm{Mn}}^{\mathrm{II}},\;5$$

(5) $$2\;\mathrm{Cu}{\left({\mathrm{ClO}}_{4}\right)}_{2}\cdot 6H_{2}O\;+\;2{\;H}_{2}\mathrm{pyaox}\;+{\;H}_{4}\mathrm{pma}\;+\;2\;\mathrm{DMF}\overset{\;H_{2}O/\mathrm{DMF}\;}{\to }{\;[{\mathrm{Cu}}_{2}\left(\mathrm{pma}\right){\left(H_{2}\mathrm{pyaox}\right)}_{2}{\left(\mathrm{DMF}\right)}_{2}]}_{n}\;+\;12{\;H}_{2}O\;+\;4{\;H}^{+}\;+\;4{\;\mathrm{ClO}}_{4}^{-}\;6$$

Series of experiments were also performed in order to investigate the influence of the electronic properties of the oximic ligand on the identity and structural properties of the isolated products; to this end, 2-methyl pyridyl ketoxime (Hmpko) was used instead of H_2_pyaox. The reaction of M(ClO_4_)_2_·6H_2_O (M = Zn, Cu), Hmpko and H_4_pma in a 4:2:1 molar ratio in DMF/H_2_O provided access to [Zn_2_(pma)(Hmpko)_2_(H_2_O)_4_]·2H_2_O (**7**·2H_2_O) and [Cu_2_(pma)(Hmpko)_2_(DMF)_2_]_n_ (**8**), according to the stoichiometric Equations (6) and (7).

(6) $$2\;\mathrm{Zn}{\left({\mathrm{ClO}}_{4}\right)}_{2}\cdot 6H_{2}O\;+\;2\;\mathrm{Hmpko}\;+{\;H}_{4}\mathrm{pma}\;\overset{\;H_{2}O/\mathrm{DMF}\;}{\to }\;[{\mathrm{Zn}}_{2}\left(\mathrm{pma}\right){\left(\mathrm{Hmpko}\right)}_{2}\left(H_{2}O{)}_{4}\right]\;\cdot 2{\;H}_{2}O\;+\;6{\;H}_{2}O\;+\;4{\;H}^{+}\;+\;4{\;\mathrm{ClO}}_{4}^{-}\;7\cdot 2H_{2}O$$

(7) $$2\;\mathrm{Cu}{\left({\mathrm{ClO}}_{4}\right)}_{2}\cdot 6H_{2}O\;+\;2\;\mathrm{Hmpko}\;+{\;H}_{4}\mathrm{pma}\;2\;\mathrm{DMF}\;\overset{\;H_{2}O/\mathrm{DMF}\;}{\to }\;[{\mathrm{Cu}}_{2}\left(\mathrm{pma}\right){\left(\mathrm{Hmpko}\right)}_{2}\left(\mathrm{DMF}{)}_{2}\right]{\;}_{n}+\;12{\;H}_{2}O\;+\;4{\;H}^{+}\;+\;4{\;\mathrm{ClO}}_{4}^{-}\;8$$

Following that, we investigated all the synthetic parameters that could lead to a different product and the deprotonation of the oximic ligand, i.e., the molar ratio of reactants, presence/kind of base, reaction conditions, metal source, etc. Indeed, by following the same experimental procedure, but by increasing the reaction time from 1 h to 4 h, green crystals of [Cu_4_(OH)_2_(pma)(mpko)_2_]_n_ (**9**) were isolated. The formation of **9** is summarized in the stoichiometric Equation (8). **9** can be also produced in good yield by reacting **8** and Cu(ClO_4_)_2_·6H_2_O in a 1:2 molar ratio, according to the stoichiometric Equation (9). Note that the OH^−^ ions in the structure of **9** come from the dissociation of H_2_O molecules.

(8) $$4\;\mathrm{Cu}{\left({\mathrm{ClO}}_{4}\right)}_{2}\cdot 6H_{2}O\;+\;2\;\mathrm{Hmpko}\;+{\;H}_{4}\mathrm{pma}\;\overset{\;H_{2}O/\mathrm{DMF}\;}{\to }{\;[{\mathrm{Cu}}_{4}{\left(\mathrm{OH}\right)}_{2}\left(\mathrm{pma}\right){\left(\mathrm{mpko}\right)}_{2}]}_{n}\;+\;22{\;H}_{2}O\;+\;8{\;H}^{+}\;+\;8{\;\mathrm{ClO}}_{4}^{-}\;9$$

(9) $${\left[{\mathrm{Cu}}_{2}\left(\mathrm{pma}\right){\left(\mathrm{Hmpko}\right)}_{2}{\left(\mathrm{DMF}\right)}_{2}\right]}_{n}\;+\;2\;\mathrm{Cu}{\left({\mathrm{ClO}}_{4}\right)}_{2}\cdot 6H_{2}O\;\overset{\;H_{2}O/\mathrm{DMF}\;}{\to }{\;[{\mathrm{Cu}}_{4}{\left(\mathrm{OH}\right)}_{2}\left(\mathrm{pma}\right){\left(\mathrm{mpko}\right)}_{2}]}_{n}\;+\;2\;\mathrm{DMF}\;+\;10{\;H}_{2}O\;+\;4{\;H}^{+}\;+\;4{\;\mathrm{ClO}}_{4}^{-}\;9$$

### 3.2. Description of Structures

Representations of the molecular structures of **1**–**9** are shown in Figure 1, Figure 2, Figure 3, Figure 4, Figure 5, Figure 6 and Figure 7 and Figures S1–S5 (Supplementary Information). Selected interatomic distances and angles are listed in Tables S1–S9.

**Figure 1:**
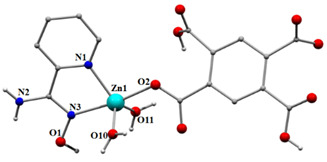
Representation of the mononuclear complex **1**. Color code: Zn, turquoise; N, navy blue; O, red; C, grey.

**1** crystallizes in the triclinic space group *P*ī. Its structure (Figure 1) consists of mononuclear [Zn(H_2_pma)(H_2_pyaox)(H_2_O)_2_] species. The coordination sphere of the metal centre is completed by one terminal *N,N*′-bidentate chelating H_2_pyaox ligand, two terminal water molecules, and one double deprotonated H_2_pma^2^^−^ ligand. Zn1 is five-coordinate adopting a distorted trigonal bipyramidal geometry (τ = 0.8) with O2 and N3 occupying the axial positions [111].

A network of intermolecular hydrogen bonding interactions stabilizes the crystal structure of **1**; this involves the protonated carboxylic groups (O4, O9), the amino group (N2), and the terminal H_2_O molecules (O10, O11) as donors, and carboxylic groups from neighboring compounds (O3, O5, O6, O7, and O8) as acceptors. Details of the metric parameters of the crystallographically established hydrogen bonds in **1** are listed in Table S10.

**Figure 2:**
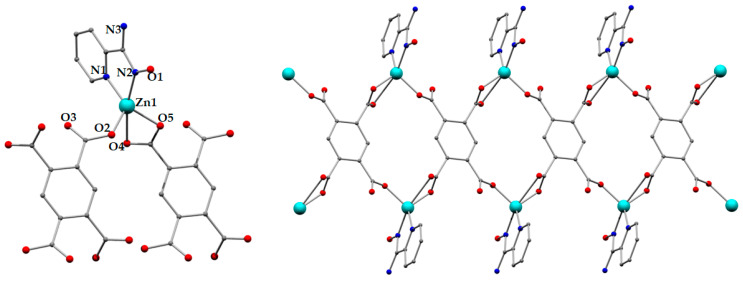
Representation of the repeating unit (**left**) and a part of the 1D double chain of **2** (**right**). Color code: Zn, cyan; N, navy blue; O, red; C, grey. The hydrogen atoms are omitted for clarity.

**2** crystallizes in the triclinic space group *P*ī; its structure (Figure 2) is a 1D double chain formed by the connection of [Zn(pma)_0.5_(H_2_pyaox)] repeating units through the μ_4_-κ*O*; κ*O*′; κ*O*″; κ*O*‴ pma^4^^−^ ligand. The coordination sphere of the metal ion is completed by two terminally ligated carboxylate groups from two different pma^4^^−^ ligands, and one neutral *N*,*N*′-bidentate chelating H_2_pyaox ligand. Zn^2+^ adopts a tetrahedral coordination geometry. Both hydrogen atoms from the NH_2_ group participate in hydrogen bonding interactions in which carboxylate groups from neighboring chains act as acceptors (N3···O3 = 2.959 Å, H1N···O3 = 2.091 Å, N3-H1N···O3 = 168.57°; N3···O4 = 3.046 Å, H2N···O4 = 2.292 Å, N3-H1N···O4 = 150.68°). The extensive hydrogen bonding interactions in **2** result in the formation of a 3D network (Figure S1 in Supplementary Materials).

**Figure 3:**
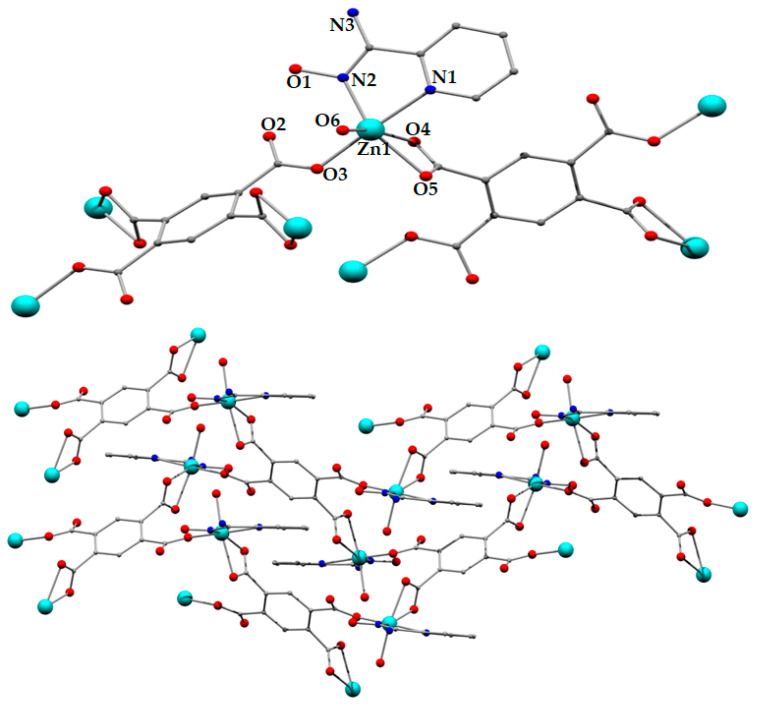
Representation of the repeating unit (**top**) and a part of the 2D chain of **3** (**bottom**). Color code: Zn^2+^, turquoise; N, navy blue; O, red; C, grey. The hydrogen atoms are omitted for clarity.

**3** crystallizes in the monoclinic space group *P2_1_/n* and is a 2D coordination polymer based on the secondary building unit [Zn(pma)_0.5_(H_2_pyaox)(H_2_O)] (Figure 3). The latter possesses a similar formula to that of the repeating unit in **2** with the main difference being the ligation of a terminal H_2_O molecule. Similar to **2**, the two oximic ligands are neutral adopting an *N*,*N*′-bidentate chelating coordination mode. The pma^4^^−^ ligands bridge four neighboring SBUs with two of the carboxylate groups adopting a chelating coordination mode, whereas the other two being terminally ligated. It is noteworthy that the different coordination mode of the pma^4-^ ligand in comparison to that in **2**, in addition to the presence of the H_2_O molecule in the coordination sphere of the metal centre, result in the Zn being six-coordinated with a distorted octahedral coordination geometry. Note that the coordination geometry of the metal ion in **2** is tetrahedral. This difference in the coordination geometry of the metal ions in **2** and **3** leads to a different orientation of the ligands around the metal ion, favoring the formation of a two-dimensional framework in **3**.

A network of hydrogen bonding interactions provide stability in the framework in **3**; these are formed between the oximic group (O1, donor) and an oxygen atom (O2, acceptor) from a carboxylate group (O1···O2 = 2.610 Å, H1A···O2 = 1.816 Å, O1-H1A···O2 = 162.55°), as well as between the terminally ligated H_2_O molecule (O6, donor) and a different carboxylate oxygen atom (O4, acceptor; O4···O6 = 2.726 Å, H6B···O4 = 2.032 Å, O6-H6B···O4 = 137.01°). 

**Figure 4:**
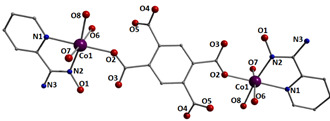
Representation of the molecular structure of the dinuclear complex **4**. Color code: Co^2+^, purple; N, navy blue; O, red; C, grey. The hydrogen atoms are omitted for clarity.

Compounds **4** and **5** display related structures with their main difference being the type of the 3d metal ion (**4**, Co^2+^; **5**, Mn^2+^). Thus, only the structure of **4** will be discussed in detail. Compound **4** crystallize in the monoclinic space group *P2_1_/n* and its structure consists of the dinuclear complex [Co_2_(pma)(H_2_pyaox)_2_(H_2_O)_6_] (Figure 4). The dinuclear molecules lie on a crystallographic inversion center with the two Co^2+^ atoms being bridged by the μ-κ*O*;κ*O*′ pma^4^^−^ ligand. The coordination sphere of each metal ion is completed by one neutral *N,N*′-bidentate chelating H_2_pyaox, and three terminal water molecules. The metal ion is six-coordinated displaying a slightly distorted octahedral geometry as a result of the relatively small bite angle of the chelating ligand (N1-Co1-N2 = 75.1(2)°). 

The crystal structure of **4** is stabilized by a strong intramolecular H bonding interaction between the oximic oxygen atom (O1) as a donor and an oxygen atom (O6) from the pma^4^^−^ ligand as an acceptor (O1···O6 = 2.533 Å, H1···O6 = 1.716 Å, O1-H1···O6 = 172.26°). A network of intermolecular hydrogen bonding interactions is formed between the terminal H_2_O molecules (O2, O3, O4) as donors and carboxylic groups from neighboring molecules as acceptors. The metric parameters of the crystallographically established, intermolecular hydrogen bonds for **4** are listed in Table S11 in the Supplementary Material.

**Figure 5:**
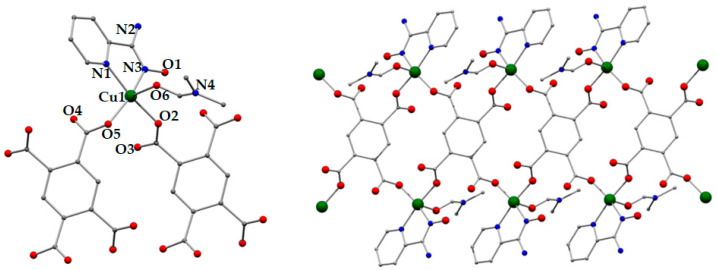
Representation of the repeating unit (**left**) and a part of the double 1D chain of **6** (**right**). Color code: Cu, blue; N, navy blue; O, red; C, grey. The hydrogens are omitted for clarity.

**6** crystallizes in the triclinic space group *P*ī; its structure (Figure 5) is based on the connection of [Cu(pma)_0.5_(H_2_pyaox)(DMF)] repeating units that result in the formation of a 1D double chain coordination polymer. The pma^4-^ ligand bridges four neighboring repeating units, adopting μ_4_- κ*O*; κ*O*′; κ*O*″; κ*O*‴ ligation mode. The coordination sphere of the metal ion is completed by two terminally ligated carboxylate groups from two different pma^4^^−^ ligands, one neutral *N*,*N*′-bidentate chelating H_2_pyaox ligand, and one terminal DMF molecule. Cu^2+^ is five-coordinate with a square pyramidal coordination geometry (τ = 0.15) with O6 from the DMF molecule occupying the axial position [111]. Both hydrogen atoms from the NH_2_ group form hydrogen bonds with oxygen atoms of carboxylate groups from neighboring chains (N2···O3 = 2.813 Å, H2N2···O3 = 1.937 Å, N2-H2N2···O3 = 176.69°; N2···O4 = 2.873 Å, H1N2···O4 = 2.065 Å, N2-H1N2···O4 = 150.10°). Furthermore, an intrachain hydrogen bond between the oximic group (O1) and an oxygen atom of a carboxylate group (O4) provide additional stability to the crystal structure (O1···O4 = 2.701 Å, H1O1···O4 = 1.921 Å, O1-H1O1···O4 = 146.29°).

Compound **7**·2H_2_O crystallizes in the triclinic group *P*ī and its crystal structure is related to that of **4**, with the main differences being: 1) the type of the 3d metal ion (**4**, Co^2+^; **7**, Zn^2+^), 2) the type of the oximic ligand (**4**, H_2_pyaox; **7**, Hmpko), and 3) the presence of two solvate H_2_O molecules in the crystal structure of **7**. The coordination sphere of the metal ions and the coordination modes of the ligands in both compounds is similar (Figure S3). The crystal structure of **7** is stabilized by two strong intramolecular H bonding interactions, which involve: 1) the oximic oxygen atom (O1) as a donor and an oxygen atom (O6) from the pma^4-^ ligand as an acceptor (O1···O6 = 2.656 Å, H1···O6 = 1.846 Å, O1-H1···O6 = 170.36°), and 2) the solvate H_2_O molecule (O9, donor) and a carboxylic oxygen atom (O6, acceptor), O9···O6 = 2.815 Å, H9B···O6 = 1.965 Å, O9-H9B···O6 = 176.96°. A network of intermolecular hydrogen bonding interactions is formed between the terminal H_2_O molecules (O2, O3, O4; donors) and carboxylic groups (O7, O8; acceptors) or the solvate H_2_O molecule (O9) from neighboring molecules. The metric parameters of the intermolecular hydrogen bonding interactions for **7** are listed in Table S12 in the Supplementary Material.

**Figure 6:**
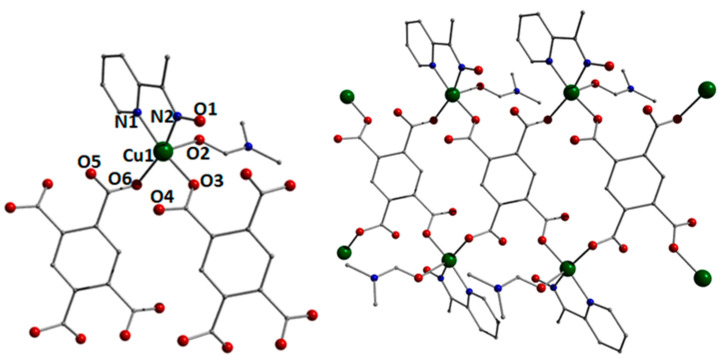
Representation of the repeating unit (**left**) and a part of the 1D double chain of **8** (**right**). Color code: Cu^II^, green; N, blue; O, red; C, grey. The hydrogen atoms are omitted for clarity.

Compound **8** crystallizes in the monoclinic space group *P2_1_/c*. Its structure consists of [Cu(pma)_0.5_(Hmpko)(DMF)]_n_ repeating units that are held together to form a 1D-double chain. (Figure 6) The coordination sphere of the Cu^2+^ atom is completed by one N,N’-bidentate chelating Hmpko ligand, one terminal DMF molecule, and two O atoms (O3 and O6) that come from two different pma^4-^ ions; the symmetry equivalent atom of O6 (O6′) links the neighboring building units forming a one-dimensional chain, whereas O3′ bridges two Cu atoms from two parallel chains, forming a 1D-double chain.

Cu1 is five-coordinate adopting a tetragonal pyramidal geometry (τ = 0.01) with O3, O6, N1, and N2 occupying the basal plane vertices and O2 the apical position [111]. There are strong intrachain hydrogen bonding interactions that stabilize the crystal structure of **8**; these are formed between the oximic group (O1, donor), and the carboxylic group of the pma^4^^−^ ion (O5), which acts as the acceptor (O1···O5 = 2.669 Å, H1O1···O5 = 1.912Å, O1-H1O1···O5 = 155.10°). The shortest Cu···Cu distance (6.5 Å) is between atoms of the same chain, whereas the shortest interchain metal···metal distance is 7.7 Å; it is noteworthy that the latter is shorter than the intrachain distance between metal atoms that belong to different chains of the 1D-double chain (8.8 Å) in **8**.

**Figure 7:**
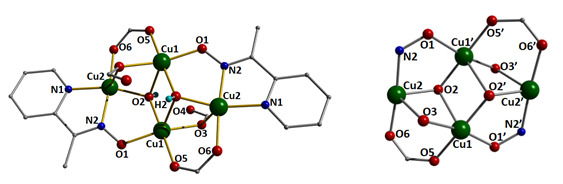
Representations of the repeating unit (**left**) in **9** and its [Cu_4_(μ_3_-OH)_2_]^6+^ butterfly” shaped core (**right**). Color code: Cu^II^, green; N, blue; O, red; C, grey. The hydrogen atoms are omitted for clarity, except from the hydrogens of the hydroxyl groups, which are cyan.

**9** crystallizes in the monoclinic space group *I*_2/a_. Its crystal structure contains a three-dimensional network based on a [Cu_4_(OH)_2_(pma)(mpko)_2_] repeating unit (Figure 7). The latter possesses a centrosymmetric “planar-butterfly” [Cu_4_(μ_3_-OH)_2_]^6+^ core with a Cu_4_ rhombus topology; the *μ*_3_-OH^-^ ions lie 0.841 Å above and below the plane formed by the four metal ions. Alternatively, the core of the SBU in **9** can be described as two face-sharing defective cubanes, i.e., with a metal atom missing from one vertex of each cubane; this motif is relatively common in metal cluster chemistry.

The coordination sphere of the metal atoms in **9** is completed by two doubly bridging anionic mpko^-^ ligands and four doubly bridging carboxylate groups; the mpko_-_ adopts the μ-κ*O*; κ*N*; κ*N*’ coordination mode. The pma- ligands link the neighboring SBUs, resulting in the formation of the 3D network, with each one bridging four Cu_4_ units. All metal atoms in **9** are five-coordinate with a tetragonal pyramidal geometry (τ = 0.28, Cu1, Cu1′; τ = 0.20, Cu2, Cu2′); O2 (for Cu1 and its symmetry equivalent) and O3 (for Cu2 and its symmetry equivalent) occupy the apical positions of the pyramids [111]. The structure of **9** is stabilized by an intramolecular hydrogen bond involving the hydroxo group (O2, donor) and one of the carboxylate groups (O4, acceptor) of the pma^4-^ ion (O2···O4 = 2.722 Å, H2···O4 = 2.113 Å, O2-H2···O4 = 133.52°), Figure S4. 

The original framework in **9** can be simplified to its underlying net following two different approaches, the so-called standard and cluster representations [112,113,114]. Applying the first one, each Cu atom and the center of the mass of the organic ligands are the nodes, which leads to a 3,4,5,8-c net with point symbol (3.4^2^)_2_(3.4^3^.5^2^)_2_(3.4^4^.5^3^.6^2^)_2_(4^12^.10^16^) [112,113,114]. This topology (Figure S5) has not been observed in the past, thus, **9** exhibits a network with an unprecedented architecture. On the other hand, following the cluster representation, in which the Cu atoms with the organic ligands are considered as a single four-coordinated node (Z_A_, Z_B_), a unimodal 4-c net is formed with **lvt** topology (Figure S6) and a (4^2^)(8^4^) point symbol.

**1**–**9** are a new family of oximic metal compounds, coordination polymers and MOFs that display unprecedented structural features. Among them, **3** and **9** are the first MOFs based on a 2-pyridyl oxime with **9** possessing a novel framework topology. **2**, **6**, and **8** join a small family of coordination polymers containing an oximic ligand [110]. The purity and stability of these compounds has been verified by pxrd studies.

### 3.3. Adsorption Studies

Thermal stability studies (Figure S17) in **9** indicated a promising adsorption potential (ca 30% mass loss below 100°C corresponding to adsorbed solvent), which prompted us to assess its metal adsorption capacity in detail. The metal encapsulation studies for **9** were carried out by soaking MOF crystals into aqueous solutions of Fe(NO_3_)_3_^.^9H_2_O. The MOF crystals were activated prior to the Fe^3+^ encapsulation in order to reduce the amount of solvent present; this was carried out by stirring the crystals in DMF for several hours and exchanging this solvent with volatile acetone, which is easily removed at 80°C. The metal encapsulation was initially investigated by batch studies using UV-vis spectroscopy. The maximum loading capacity obtained for Fe^3+^ in the case of Fe(NO_3_)_3_.9H_2_O as a metal source is 104 mg Fe^3+^/g **9** (1.50 mol Fe^3+^/mol **9**).

**Figure 8:**
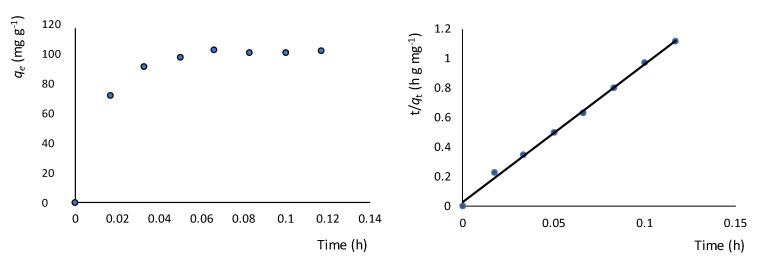
Left: metal adsorption capacity (mg g^−1^) versus time (h) plot; right: simulation of the experimental data to the pseudo-second order kinetic model. The solid lines represent the fitting of the data.

The Fe^3+^ adsorption by **9** exhibits fast kinetics; there is a smooth increase in the adsorption capacity over time, which after 5 min reaches a plateau (Figure 8, left). No metal adsorption is observed after 5 min. In order to get a better insight into the adsorption mechanism, the experimental kinetic data were fitted to a theoretical model; pseudo-first order and pseudo-second order kinetic models were used according to Equations (10) and (11), respectively [115,116,117].

(10) $$\ln\left(q_{e}-{\;q}_{t}\right)=\ln q_{e}\;-{\;k}_{1}t$$

(11) $$\frac{t}{q_{t}}=\frac{1}{k_{2}q_{e}{}^{2}}+\frac{1}{q_{e}}t$$

where k_1_ and k_2_ are the rate constants for the pseudo-first and pseudo-second kinetic models, respectively. A good fit was obtained for the pseudo-second kinetic model (Figure 8, right), which is indicative of a chemisorption mechanism, i.e., the formation of a strong interaction between the encapsulated Fe^3+^ and **9**. The corresponding fitting parameters are *R*^2^ = 0.9968, *q_e_* = 107.83 mg Fe^3+^/g **9**, which is in very good agreement with the experimental data.

**Figure 9:**
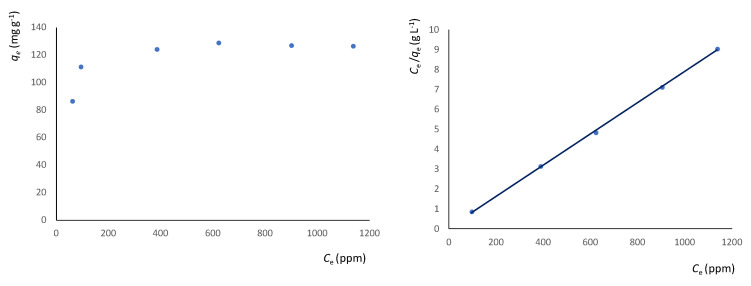
Left: Equilibrium data for the metal adsorption by **9** (contact time: 20 min); right: fitting of the metal adsorption data to the Langmuir model.

The impact of the Fe^3+^ source, and in particular the impact of the kind of counterion, on the kinetic and/or thermodynamic properties of the metal encapsulation by **9** was also studied by using FeCl_3_ instead of Fe(NO_3_)_3_. It was found that the maximum loading capacity is 105 mg Fe^3+^/g **9** (1.52 mol Fe^3+^/mol **9**) and is reached after 7 min stirring. The metal adsorption equilibrium data are plotted in Figure 9. The best fitting of the data is provided by the Langmuir model [118,119], considering a monolayer adsorption with a finite number of homogeneous and equivalent active sites, Equation (12):

(12) $$\frac{c_{e}}{q_{e}}\;=\;\frac{c_{e}}{q_{s}}+\frac{1}{q_{s}K_{L}}$$

where *q_e_* (mg/g) is the amount of metal ion per gram of **9** at the equilibrium concentration *C_e_* (ppm of metal ion remaining in solution), *q_s_* is the maximum adsorption capacity of **9**, and *K_L_* is the Langmuir constant; the fitting parameters are *q_s_* = 129.87 mg Fe/g **9**, *K_L_* = 5 × 10^2^ (L mol^−1^) and *R*^2^ = 0.9996. The corresponding kinetic plot is shown in Figure S8. Hence, the nature of the counterion of the Fe^3+^ source does not affect the metal encapsulation capacity of **9**. Other metal ions were also tested, including Co^2+^, Ni^2+^, Mn^2+^, and Cr^3+^; however, no adsorption was observed even after one day stirring, indicating that **9** exhibits high selectivity for Fe^3+^. 

**Figure 10:**
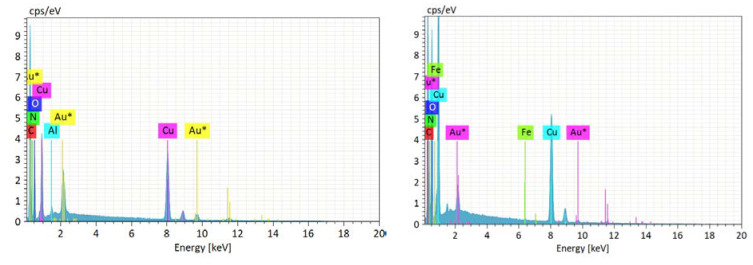
EDX spectra of **9** (**left**) and Fe@**9** (**right**).

The Fe^3+^ adsorption capacity of **9** was further investigated by EDX studies. Figure 10 shows the EDX spectra of **9** and Fe@**9**; Cu, C, and O are detected in both samples, with the the second sample displaying one additional peak corresponding to the Fe^3+^ ion that has been adsorbed, hence confirming the metal uptake. It is noteworthy that **9** can be easily regenerated from Fe@**9** by treatment with a 0.2 M EDTA solution. The activated MOF displays an identical sorption capacity with that of the original **9**, which is retained for three cycles of regeneration/reuse experiments (Figure S9). Thus, **9** is reusable, a property which is paramount for practical applications.

The good Fe^3+^ adsorption capacity of **9** prompted us to evaluate its encapsulation performance for other species. 6-methyluracil is a compound for which the development of new sorbents or sensors is of great importance; it is a key component of many drugs, including anti-ulcer agents, radiation protective agents, and immunological adjuvants which help enhance immune responses, but on the other hand, it can also be encountered as a toxic byproduct in the synthesis of biologically important DNA adducts. If small impurities of such byproducts are not identified and removed, it could have devastating consequences for the corresponding biological assays and medical treatments. Thus, the development of efficient carriers/sorbents for the delivery or removal of these species would provide new insight into drug manufacturing. Considering also the fact that MOFs have not yet been used for drug purification, this would expand the range of potential applications of MOFs. With the above in mind, and following a similar process to the one used for metal adsorption, the 6-methylyracil capacity of **9** was investigated. This is an ongoing project, but preliminary results (Figure S10) indicate that **9** possess a good performance in 6-methyluracil adsorption; the maximum uptake capacity is 167 mg 6-methyluracil/g **9** for a 1:1 ratio 6-methyluracil:**9** in an aqueous solution. It exhibits fast kinetics with the maximum encapsulation being reached after 6 min stirring. It is noteworthy, though, that the regeneration of the MOF after the 6-methyluracil adsorption is not feasible. These initial results show the potential of **9**, and MOFs in general, to be used in purification processes during drug manufacturing. Investigations are now in progress to assess the selectivity of **9** for 6-methyluracil and other toxic byproducts, which is a requirement for the use of MOFs in such applications.

### 3.4. Magnetism Studies

**Figure 11:**
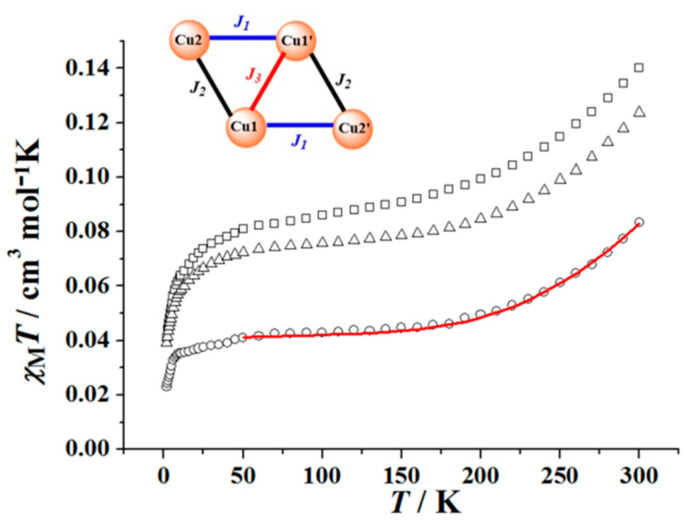
χ_M_*T* vs. *T* plot for **9** (circles) Fe@**9**-1 (triangles) and Fe@**9**-2 (squares). Solid line represents the best fit for **9**. Inset: the exchange interactions in **9**.

Solid state DC magnetic susceptibility measurements were performed on a polycrystalline sample of **9** in an applied field 0.03 T and temperature range 2–300 K. The obtained data are shown as a χ_M_*T* vs. *T* plot in Figure 11. The χ_M_*T* value at 300 K is 0.075 cm^3^·mol^−1^ K, appreciably lower than the spin-only (*g* = 2) value of 1.5 cm^3^·mol^−1^ K expected for four non-interacting Cu^2+^ centres (*S* = 1/2), revealing strong antiferromagnetic coupling within the framework SBU. This is further supported by the overall profile of the plot, which is indicative of strong antiferromagnetic interactions and a diamagnetic ground state for **9**.

The isotropic Heisenberg spin Hamiltonian for **9** is given by Equation (13), where *J*_1_ is associated with the Cu1…Cu2′ interactions through a diatomic oximato group; *J*_2_ is associated with the Cu1…Cu2 interaction through one monoatomic carboxylate bridge; and *J*_3_ describes the coupling of Cu1…Cu1′ through a *μ*_3_-OH^−^ group (Figure 11).

ℋ = −2*J*_1_ (*Ŝ*_Cu1_·*Ŝ*_Cu2′_ + *Ŝ*_Cu1′_·*Ŝ*_Cu2_)-2*J*_2_(*Ŝ*_Cu1_·*Ŝ*_Cu2_+ *Ŝ*_Cu1′_·*Ŝ*_Cu2′_) −2*J*_3_(*Ŝ*_Cu1·_*Ŝ*_Cu1′_) (13)

The fitting of the experimental data to the Hamiltonian was performed using the PHI software [120] and is shown with a solid line in Figure 10; the fitting parameters are *J*_1_ = −508 cm^−1^, *J*_2_ = −1.45 cm^−1^, *J*_3_ = −8.77 cm^−1^, and *g* = 2.07. *J*_1_ is significantly stronger than *J*_2_ and *J*_3_; this high value of antiferromagnetic coupling is common in complexes containing oximes and Cu ions; such complexes often exhibit diamagnetic behavior [82]. *J*_2_ and *J*_3_ are too small to take them as reliable values because at the temperature they start to operate, the complex is diamagnetic due to the extremely high value of *J*_1_. 

An initial investigation of the potential of magnetism to be used for the development of novel MOF-based sensors was performed by exploring the impact of the encapsulated metal ion on the magnetic properties of **9**. To this end, DC magnetic susceptibility studies were performed on the polycrystalline samples of Fe@**9**-1 and Fe@**9**-2, where Fe@**9**-1 is the Fe@**9** aggregate after 1 min stirring, during which **9** is expected to have reached ca. its half metal encapsulation capacity, and Fe@**9**-2 is the aggregate at its maximum metal adsorption capacity. A closer inspection in Figure 11 shows that, although the overall profile of the three plots is similar, the increase of the paramagnetic component in the MOF pores have an effect on the observed χ_M_*T* value at room temperature; the latter increases from 0.073 cm^3^·mol^−1^ K (for **9**) to 0.115 cm^3^·mol^−1^ K (for Fe@**9**-1) and 0.135 cm^3^·mol^−1^ K (for Fe@**9**-2), indicating that the magnetic properties of the Fe@**9** aggregate are affected by the amount of metal encapsulated in the MOF pores.

## 4. Conclusions

The initial employment of a 2-pyridyl oxime (pyridine-2 amidoxime, H_2_pyaox; 2-methyl pyridyl ketoxime, Hmpko) in combination with 1,2,4,5-benzene tetracarboxylic acid (pyromellitic acid), H_4_pma, provided access to nine new compounds with interesting structural features, paving the way to the development of an alternative synthetic route towards new MOFs and coordination polymers. [Zn_2_(pma)(H_2_pyaox)_2_(H_2_O)_2_]_n_ (**3**) and [Cu_4_(OH)_2_(pma)(mpko)_2_]_n_ (**9**) are the first examples of MOFs based on a 2-pyridyl oxime, whereas [Zn_2_(pma)(H_2_pyaox)_2_]_n_ *(***2**), [Cu_2_(pma)(H_2_pyaox)_2_(DMF)_2_]_n_ (**6**), and [Cu_2_(pma)(Hmpko)_2_(DMF)_2_]_n_ (**8**) are new members of a small family of coordination polymers bearing this type of ligands. **9** has a novel 3,4,5,8-c net topology and is based on a butterfly-shaped Cu_4_ SBU. DC magnetic susceptibility studies revealed that there are strong antiferromagnetic interactions between the metal centers in **9**, which lead to a diamagnetic ground state.

The metal encapsulation capacity of **9** was tested for a variety of different metal ions and showed that it exhibits selectivity for Fe^3+^ adsorption. It is noteworthy that the magnetic properties of **9** depend on the amount of Fe^3+^ present in to the MOF pores, revealing that magnetism can be an alternative technique for the detection of environmentally hazardous chemicals, and this can be especially useful for species that do not affect the photoluminescence properties or the color of a compound, hence they cannot be detected by the commonly used sensors. Treatment of the Fe@**9** aggreagate with a 0.2 M EDTA solution leads to the removal of the metal ions from the MOF pores and the regeneration of the latter. **9** retains its metal adsorption capacity for three cycles of regeneration/reuse experiments.

## Figures and Tables

**Table 1:** Crystallographic data for complexes **1**–**9**.

**Complex**	**1**	**2**	**3**
Empirical formula	C_16_H_15_N_3_O_11_Zn	C_11_H_8_N_3_O_5_Zn	C_11_H_10_N_3_O_6_ Zn
Formula weight	490.68	327.57	346.60
Crystal system	Triclinic	Triclinic	Monoclinic
Space group	*P*ī	*P*ī	*P2_1_/n*
*a* (Å)	8.1151(3)	6.9864(7)	11.4913(8)
*b* (Å)	9.8550(4)	8.9530(8)	6.6261(5)
*c* (Å)	11.8506(5)	10.4524(9)	16.1381(12)
*α* (^o^)	93.283(3)	83.697(7)°	90
*β* (^o^)	105.135(3)	94.887(2)	104.335(8)°
*γ* (^o^)	92.769(3)	71.582(9)°	90
*V* (Å^3^)	911.40(6)	612.98(10)	1190.54(16)
*Ζ*	2	2	4
*ρ*_calc_ (g cm^−3^)	1.788	1.775	1.934
Radiation, *λ* (Å)	0.71073	1.54184	0.71073
μ (mm^−1^)	1.420	3.042	2.099
Temperature (K)	297.9(6)	100(2)	100(2)
Measd/independent reflns (*R*_int_)	4250/3667 (0.0439)	3860/2400 (0.0451)	4714/2088 (0.0371)
Parameters refined	316	197	197
GoF (on *F*^2^)	1.059	1.078	1.055
*R*_1_^a^ (*I* > 2*σ*(*Ι*))	0.0352	0.0517	0.0353
*wR*_2_^b^ (*I* > 2*σ*(*Ι*))	0.0834	0.1377	0.0879
(Δ*ρ*)_max_/(Δ*ρ*)_min_ (e Å^−3^)	0.572/−0.504	2.839/−0.831	0.573/−0.632
**Complex**	**4**	**5**	**6**
Empirical formula	C_22_H_28_N_6_O_16_Co_2_	C_22_H_28_N_6_O_16_Mn_2_	C_14_H_15_N_4_O_6_Cu
Formula weight	750.36	742.38	398.84
Crystal system	Monoclinic	Monoclinic	Triclinic
Space group	*P2_1_/n*	*P2_1_/n*	Pī
*a* (Å)	10.9732(9)	11.1773(8)	6.5039(4)
*b* (Å)	11.7514(8)	12.2116(8)	10.0104(9)
*c* (Å)	11.6014(13)	11.5293(9)	12.7604(9)
*a* (^o^)	90	90	96.743(6)
*β* (^o^)	107.619(10)	106.542(7)	91.660(5)
*γ (^o^)*	90	90	108.375(7)
*V* (Å^3^)	1425.8(2)	1508.5(2)	781.05(11)
*Ζ*	2	2	2
*ρ*_calc_ (g cm^−3^)	1.748	1.634	1.696
Radiation, *λ* (Å)	1.54184	0.71073	0.71073
μ (mm^−1^)	9.917	0.921	1.440
Temperature (K)	100(2)	298.0(2)	299.0(1)
Measd/independent reflns (*R*_int_)	5285/2830 (0.0324)	3603/2795 (0.0680)	3616/2040 (0.1137)
Parameters refined	212	244	240
GoF (on *F*^2^)	1.112	1.047	0.873
*R*_1_^a^ (*I* > 2*σ*(*Ι*))	0.0547	0.0479	0.0526
*wR*_2_^b^ (*I* > 2*σ*(*Ι*))	0.1500	0.1076	0.0868
(Δ*ρ*)_max_/(Δ*ρ*)_min_ (e Å^−3^)	0.586/−0.602	0.825/−0.399	0.823/−0.678
**Complex**	**7**∙2H_2_O	**8**	**9**
Empirical formula	C_24_H_34_N_4_O_18_Zn_2_	C_30_H_32_N_6_O_12_Cu_2_	C_12_H_9_N_2_O_6_Cu_2_
Formula weight	797.29	795.69	404.29
Crystal system	Triclinic	Monoclinic	Monoclinic
Space group	*P*ī	*P2_1_/c*	*I2/a*
*a* (Å)	7.1623(7)	6.4617(3)	16.3272(7)
*b* (Å)	8.3966(8)	25.5232(9)	10.4575(4)
*c* (Å)	13.4476(10)	10.1083(5)	15.0472(7)
*a (^o^)*	106.056(8)	90	90
*β* (^o^)	104.078(8)	106.321(5)	102.188(5)
*γ (^o^)*	90.547(8)	90	90
*V* (Å^3^)	751.27(12)	1599.91(13)	2511.27(19)
*Ζ*	1	2	8
*ρ*_calc_ (g cm^−3^)	1.762	1.652	2.139
Radiation, *λ* (Å)	1.54184	0.71073	1.54184
μ (mm^−1^)	2.785	1.404	4.522
Temperature (K)	100(2)	299.0(2)	100(2)
Measd/independent reflns (*R*_int_)	4859/2943 (0.0323)	3877/2762 (0.0754)	4290/2480 (0.0297)
Parameters refined	225	233	204
GoF (on *F*^2^)	1.087	0.954	1.097
*R*_1_^a^ (*I* > 2*σ*(*Ι*))	0.0638	0.0433	0.0501
*wR*_2_^b^ (*I* > 2*σ*(*Ι*))	0.1890	0.0978	0.1471
(Δ*ρ*)_max_/(Δ*ρ*)_min_ (e Å^−3^)	0.957/−1.077	0.450/−0.496	1.587/−1.066

^a^*R*_1_ = Σ(|*F*_o_| − |*F*_c_|)/Σ(|*F*_o_|). ^b^ *wR*_2_ = {Σ[*w*(*F*_o_^2^ − *F*_c_^2^)^2^]/Σ[*w*(*F*_o_^2^)^2^]}^1/2^.

## Supplementary Materials

The following are available online at https://www.mdpi.com/1996-1944/13/18/4084/s1, Figure S1: Representation of the 3D network formed through hydrogen bonding interactions in **2**. Figure S2: Representation of the molecular structure of the dinuclear complex **5**. Figure S3: Representation of the molecular structure of the dinuclear complex **7**. Figure S4: Representation of the intramolecular hydrogen bonding interactions in **9**. Figure S5: Representation of the underlying 3,4,5,8-coordinated net in the standard representation in **9**. Figure S6: The underlying net in the cluster representation with the **lvt** topology with Point Symbol (4^2^)(8^4^) in **9**. Figure S7: Comparison of the theoretical and experimental pxrd pattern for **9**. Figure S8: Left: metal adsorption capacity (mg g^−1^) versus time (h) plot for the encapsulation of FeCl_3_ by **9**; right: simulation of the experimental data to the pseudo-second order kinetic model. The solid lines represent the fitting of the data. The corresponding fitting parameters are *R*^2^ = 0.9937, *q*_e_ = 103.09 mg Fe^3+^/g **9**, in very good agreement with the experimental data. Figure S9: Comparison of the pxrd pattern of the initial **9** with that of the regenerated material. Figure S10: UV studies of the 6-methyluracil adsorption by **9**; (a) 0.05 mmol **9**/0.1 mmol 6-methyluracil and (b) 0.1 mmol **9**/0.1 mmol 6-methyluracil (in 10 mL of water). Tables S1–S9: Selected interatomic distances (Å) and angles for **1**–**9**. Table S10: Hydrogen bonding details for **1**. Table S11: Intermolecular hydrogen bonding details for **4**^.^ Table S12: Intermolecular hydrogen bonding details for **7**.
