# Supplementary material for: From 1D Coordination Polymers to Metal Organic Frameworks by the Use of 2-Pyridyl Oximes
Source: Materials (Basel). 2020 Sep 14;13(18):4084. doi: 10.3390/ma13184084 (PMC7560365; doi:10.3390/ma13184084)
Supplement: Supplementary file 1 [file materials-13-04084-s001.pdf]

materials

Supplementary Material

From 1D Coordination Polymers to Metal Organic Frameworks by the Use of 2-Pyridyl Oximes

Ioannis Mylonas-Margaritis [1,], Auban Gérard [1], Katerina Skordi [2], Julia Mayans [3], Anastasios Tasiopoulos [2], Patrick McArdle [1] and Constantina Papatriantafyllopoulou [1,*]

[1] School of Chemistry, College of Science and Engineering, National University of Ireland Galway, University Road, H91 TK33 Galway, Ireland; SSPC, Synthesis and Solid State Pharmaceutical Centre, Ireland; i.mylonas-margaritis1@nuigalway.ie (I.M.-M.); auban.gerard@uha.fr (A.G.); patrick.mcardle@nuigalway.ie (P.M.)

[2] Department of Chemistry, University of Cyprus, 1678 Nicosia, Cyprus; skordi.katerina@ucy.ac.cy (K.S.); atasio@ucy.ac.cy (A.T.);

[3] Instituto de Ciencia Molecular (ICMol), Universidad de Valencia, Catedrático José Beltran 2, 46980 Paterna (Valencia), Spain; julia.mayans@qi.ub.edu

* Correspondence: constantina.papatriantafyllopo@nuigalway.ie; Tel.: +353-91-493462

Received: 14 August 2020; Accepted: 7 September 2020; Published:

Table S1. Selected interatomic distances (Å) and angles for **1**.

Bonds			
Zn1-N1	2.059(2)	Zn1-O11	1.987(2)
Zn1-N3	2.139(2)	Zn1-O2	2.049(2)
Zn1-O10	1.995(2)		
Angles			
O10-Zn1-O2	91.54(8)	O2-Zn1-N3	172.22(7)
O10-Zn1-O11	121.30(8)	O2-Zn1-N1	95.54(7)
O10-Zn1-N3	91.78(8)	O11-Zn1-N3	90.93(8)
O10-Zn1-N1	111.61(8)	O11-Zn1-N1	125.97(8)
O2-Zn1-O11	93.34(7)	N1-Zn1-N3	76.69(8)

Table S2. Selected interatomic distances (Å) and angles for **2**.

Bonds			
Zn1-O2	1.954(3)	Zn1-N2	2.031(4)
Zn1-N1	2.019(3)	Zn1-O5	1.966(4)
Angles			
O2-Zn1-O5	98.2(1)	N1-Zn1-O5	113.4(1)
O2-Zn1-N1	127.9(1)	N1-Zn1-N2	80.1(1)
O2-Zn1-N2	108.6(1)	N2-Zn1-O5	126.1(1)

Table S3. Selected interatomic distances (Å) and angles for **3**.

Bonds			
Zn1-O6	2.056(3)	Zn1-N2	2.058(3)
Zn1-O5	2.228(2)	Zn1-N1	2.198(2)
Zn1-O4	2.292(2)	Zn1-O3	2.027(2)
Angles			
O5-Zn1-O3	88.44(9)	O6-Zn1-O3	82.62(1)
O3-Zn1-N2	108.1(1)	O6-Zn1-N2	96.88(1)
N2-Zn1-N1	75.4(1)	O4-Zn1-N2	97.76(9)
N1-Zn1-O5	88.99(9)	O4-Zn1-N1	84.47(9)

O6-Zn1-N1	95.33(1)	O4-Zn1-O5	58.28(8)
O6-Zn1-O5	106.54(9)	O4-Zn1-O3	96.64(9)

Table S4. Selected interatomic distances (Å) and angles for **4**.

Bonds			
Co1-N2	2.100	Co1-O3	2.132
Co1-O2	2.110	Co1-O4	2.070
Co1-O5	2.089	Co1-N1	2.162
Angles			
N1-Co1-O2	89.92(1)	N2-Co1-O4	93.9(1)
O2-Co1-O5	83.74(9)	N2-Co1-N1	75.1(1)
O5-Co1-O4	94.8(1)	O3-Co1-O4	83.7(1)
O4-Co1-N1	92.2(1)	O3-Co1-N1	95.8(1)
N2-Co1-O2	97.6(1)	O3-Co1-O2	84.78(9)
N2-Co1-O5	101.9(1)	O3-Co1-O5	87.5(9)

Table S5. Selected interatomic distances (Å) and angles for **5**.

Bonds			
Mn1-O6	2.149(3)	Mn1-O2	2.136(2)
Mn1-N1	2.304(3)	Mn1-N3	2.244(2)
Mn1-O8	2.170(2)	Mn1-O7	2.191(2)
Angles			
N3-Mn1-O2	97.69(8)	O7-Mn1-O6	82.3(1)
N3Mn1-O8	99.04(8)	O7-Mn1-N1	94.45(9)
N3-Mn1-N1	70.46(8)	O6-Mn1-O2	95.4(1)
N3-Mn1-O6	94.7(1)	O6-Mn1-N1	92.0(1)
O7-Mn1-O8	83.63(9)	O2-Mn1-O8	85.79(8)
O7-Mn1-O2	97.66(8)	O8-Mn1-N1	89.74(8)

Table S6. Selected interatomic distances (Å) and angles for **6**.

Bonds			
Cu1-O6	2.307(3)	Cu1-N1	1.992(4)
Cu1-O2	1.926(3)	Cu1-N3	1.970(4)
Cu1-O5	1.961(3)		
Angles			
O5-Cu1-O2	90.9(1)	O6-Cu1-O2	88.0(1)
O2-Cu1-N3	93.5(1)	O6-Cu1-N3	101.8(1)
N3-Cu1-N1	79.2(2)	O6-Cu1-N1	90.2(1)
N1-Cu1-O5	97.0(1)	O6-Cu1-O5	95.1(1)

Table S7. Selected interatomic distances (Å) and angles for **7**.

Bonds			
Zn1-O4	2.062(3)	Zn1-N1	2.056(4)
Zn1-O2	2.100(3)	Zn1-O5	2.019(4)
Zn1-N2	2.041(4)	Zn1-O3	2.100(3)
Angles			
O4-Zn1-N2	91.1(1)	O2-Zn1-O5	85.0(1)
O4-Zn1-N1	91.1(1)	O2-Zn1-O3	89.7(1)
O4-Zn1-O5	91.9(1)	N2-Zn1-O5	103.8(1)
O4-Zn1-O3	92.2(1)	O5-Zn1-O3	83.0(1)
O2-Zn1-N2	87.4(1)	O3-Zn1-N1	94.5(1)
O2-Zn1-N1	92.0(1)	N1-Zn1-N2	78.5(1)

Table S8. Selected interatomic distances (Å) and angles for **8**.

Bonds			
Cu1-N1	1.972(3)	Cu1-O3	1.903(3)
Cu1-N2	2.032(3)	Cu1-O6	1.965(2)
Cu1-O2	2.269(2)		
Angles			
O2-Cu1-N1	88.6(1)	N2-Cu1-O3	93.2(1)
O2-Cu1-N2	100.7(1)	O3-Cu1-O6	91.75(9)
O2-Cu1-O3	88.63(9)	O6-Cu1-N1	97.2(1)
O2-Cu1-O6	99.58(8)	N2-Cu1-N1	78.8(1)

Table S9. Selected interatomic distances (Å) and angles for **9**.

Bonds			
Cu1-O1	1.943(3)	Cu2-O2	1.906(3)
Cu1-O2	1.920(3)	Cu2-O3	2.410(3)
Cu1-O2	2.334(3)	Cu2-O6	1.960(3)
Cu1-O3	1.958(3)	Cu2-N2	1.984(4)
Cu1-O5	1.961(3)	Cu2-N1	1.988(4)
Angles			
N2-Cu2-O2	87.2(1)	O2-Cu2-O3	82.4(1)
N2-Cu2-O3	90.4(1)	Cu2-O2-Cu1	94.2(1)
N2-Cu2-O6	174.3(2)	Cu2-O3-Cu1	90.5(1)
N2-Cu2-N1	80.4(2)	O3-Cu1-O5	89.6(1)
O1-Cu1-O2	92.0(1)	O3-Cu1-O2	83.3(1)

Table S10. Hydrogen bonding details for **1**[a].

D–H···A	D···A (Å)	H···A (Å)	DHA (º)	Symmetry Operator of A
O(1)–H(1O4)···O(8)	2.693	1.839	165.12	$x, -1+y, z$
N(2)–H(2N2)···O(5)	3.069	2.399	135.16	$x, -1+y, 1+z$
O(10)–H(10A)···O(7)	2.729	1.870	170.65	$x, y, -1+z$
O(9)–H(1O9)···O(6)	2.577	1.713	166.14	$1-x$, 1-y, $1-z$
O(11)–H(11B)···O(3)	2.673	1.831	161.60	$1-x$, 1-y, $2-z$

[a] A = acceptor, D = donor

Table S11. Intermolecular hydrogen bonding details for **4**[a].

D–H···A	D···A (Å)	H···A (Å)	DHA (º)	Symmetry operator of A
O(2)–H(2B)···O(7)	2.643	1.804	162.64	$-1+x, y, z$
O(3)–H(3B)···O(6)	2.711	1.667	166.11	$-1+x, y, z$
O(2)–H(2A)···O(4)	2.936	2.326	126.06	$-1/2+x, ½-y, ½+z$
O(4)–H(4B)···O(6)	2.656	1.646	155.62	$-1/2+x, ½-y, ½+z$

[a] A = acceptor, D = donor

Table S12. Intermolecular hydrogen bonding details for **7**[a].

D–H···A	D···A (Å)	H···A (Å)	DHA (º)	Symmetry operator of A
O(3)–H(3B)···O(7)	2.759	1.909	166.16	$-1+x, -1+y, z$
O(2)–H(2A)···O(8)	2.602	1.739	178.63	$-1+x, -1+y, z$
O(2)–H(2B)···O(9)	2.876	2.066	151.29	$x, -1+y, z$
O(4)–H(4B)···O(8)	2.674	1.860	157.81	$-1/2+x, ½-y, ½+z$

[a] A = acceptor, D = donor

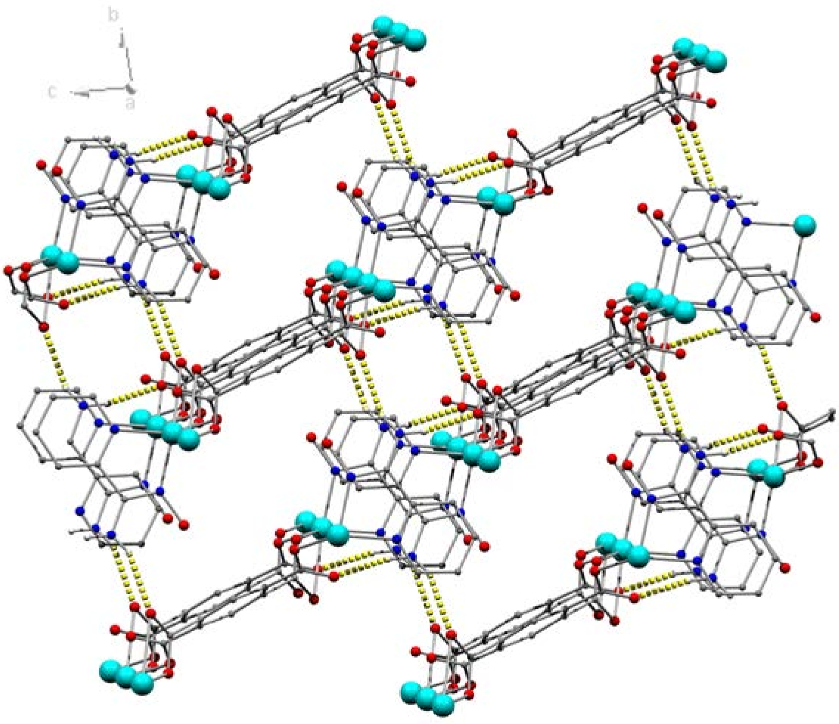

Figure S1. Representation of the 3D network formed through hydrogen bonding interactions in **2**. Colour code: Zn, turquoise; N, navy blue; O, red; C, grey.

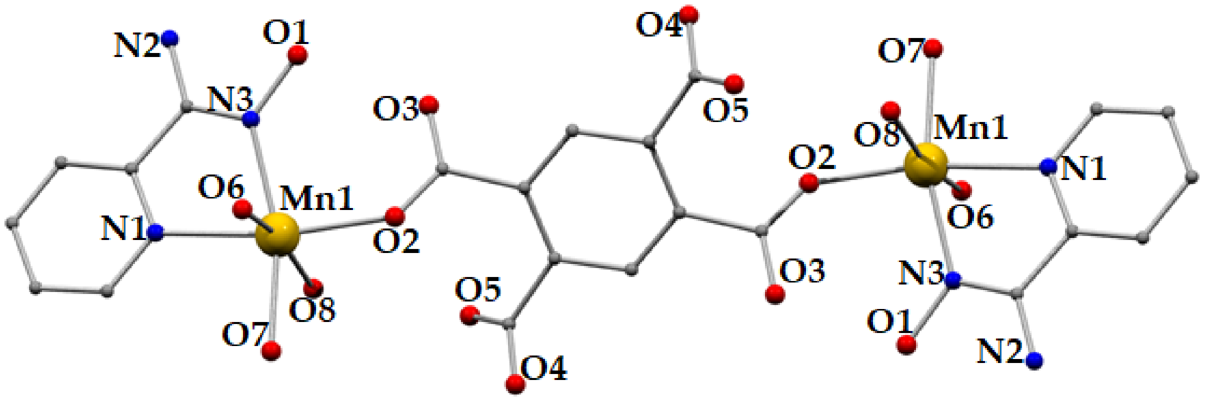

Figure S2. Representation of the molecular structure of the dinuclear complex **5**. Colour code: Mn^{II}, yellow; N, navy blue; O, red; C, grey. The hydrogen atoms are omitted for clarity.

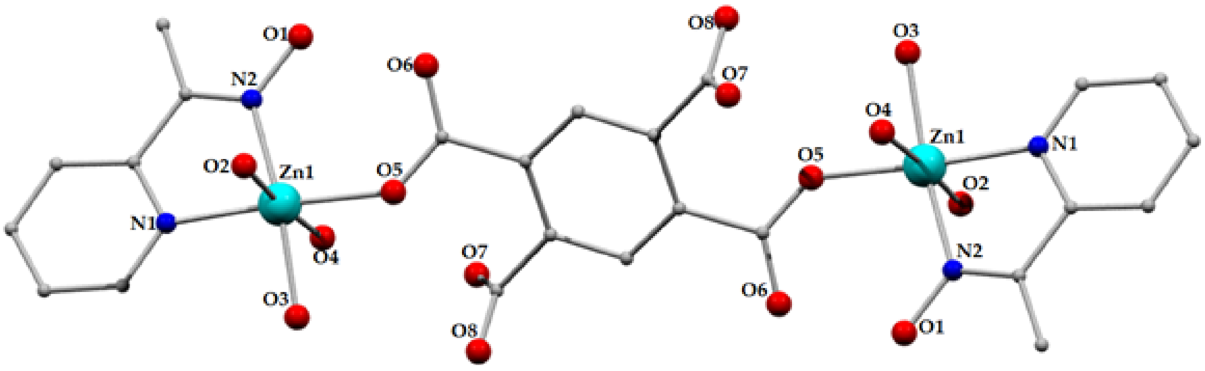

Figure S3. Representation of the molecular structure of the dinuclear complex **7**. Colour code: Zn^{II}, cyan; N, navy blue; O, red; C, grey. The hydrogen atoms are omitted for clarity.

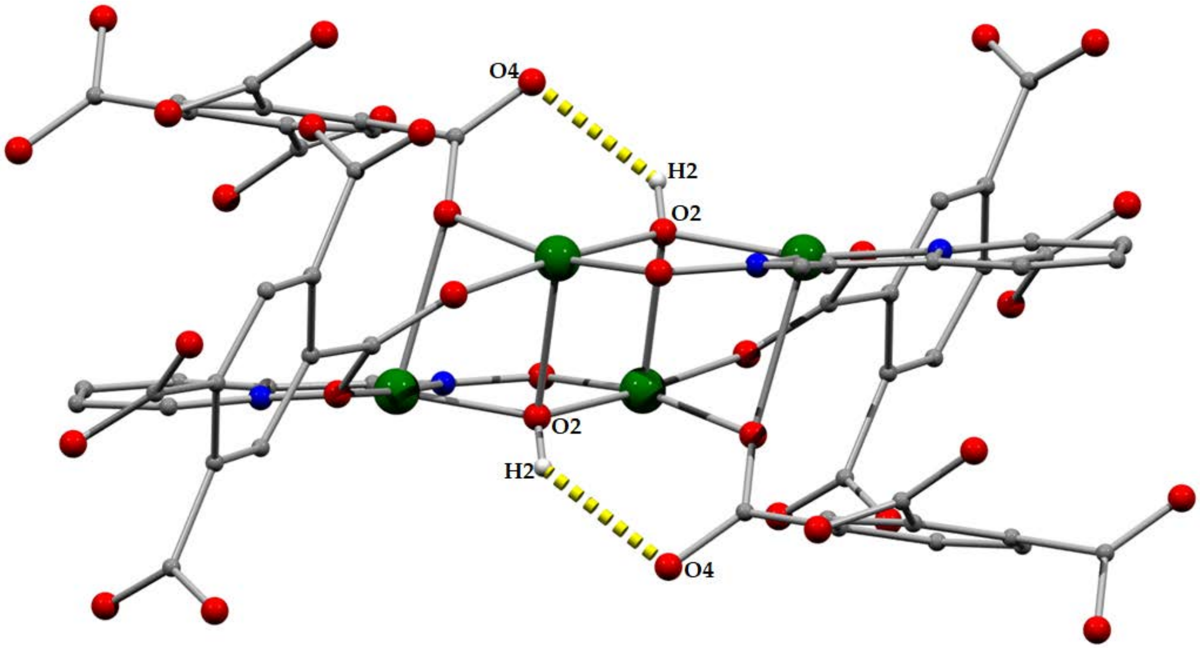

Figure S4. Representation of the intramolecular hydrogen bonding interactions in **9**.

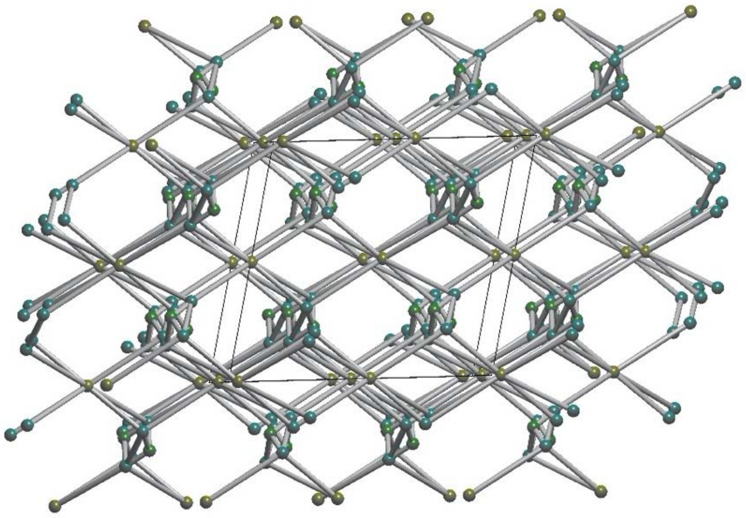

Figure S5. Representation of the underlying 3,4,5,8-coordinated net in the standard representation in **9**.

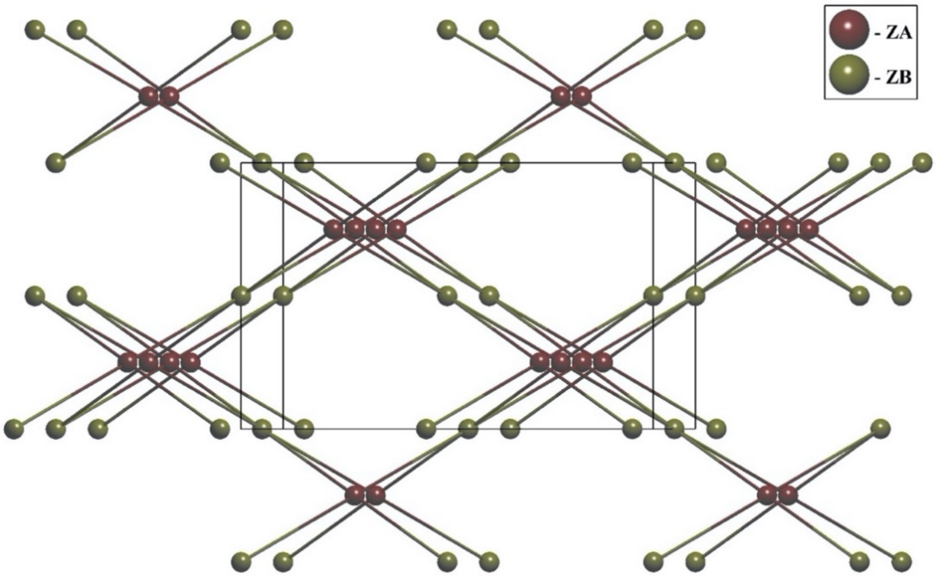

Figure S6. The underlying net in the cluster representation with the **lvt** topology with Point Symbol $(4^2)(8^4)$ in **9**.

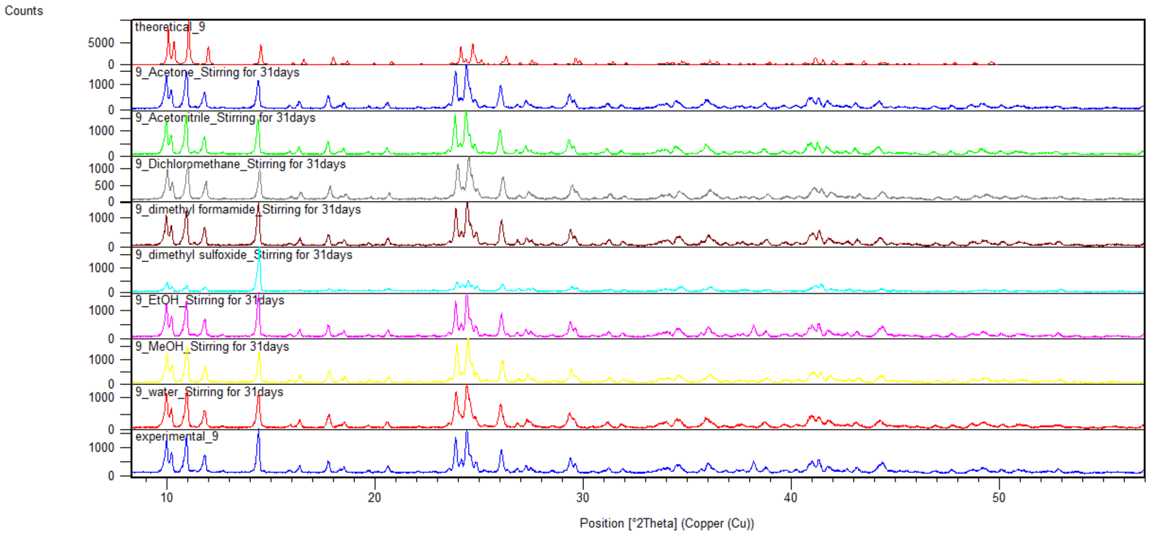

Figure S7. Comparison of the theoretical and experimental pxrd pattern for **9**.

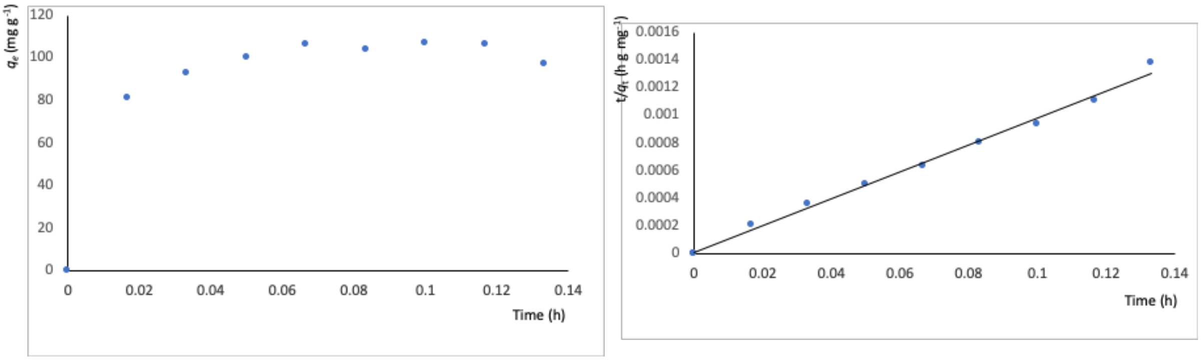

Figure S8. Left: metal adsorption capacity (mg g^{-1}) versus time (h) plot for the encapsulation of $FeCl_3$ by **9**; right: simulation of the experimental data to the pseudo-second order kinetic model. The solid lines represent the fitting of the data. The corresponding fitting parameters are $R^2 = 0.9937$, $q_e = 103.09$ mg Fe^{3+}/g **9**, in very good agreement with the experimental data.

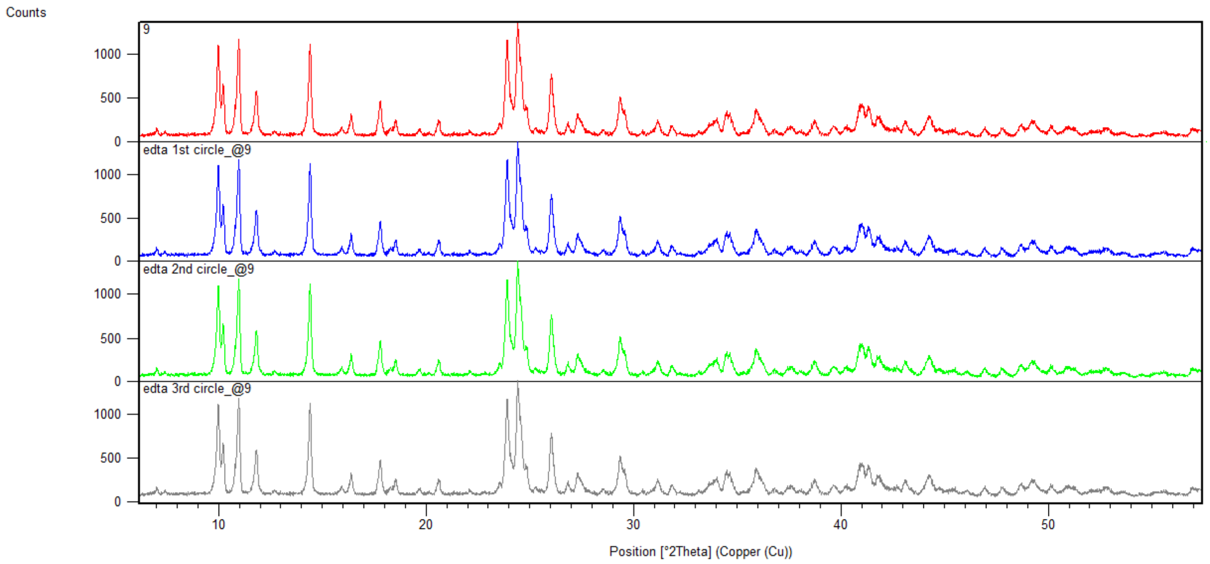

Figure S9. Comparison of the pxrd pattern of the initial **9** with that of the regenerated material.

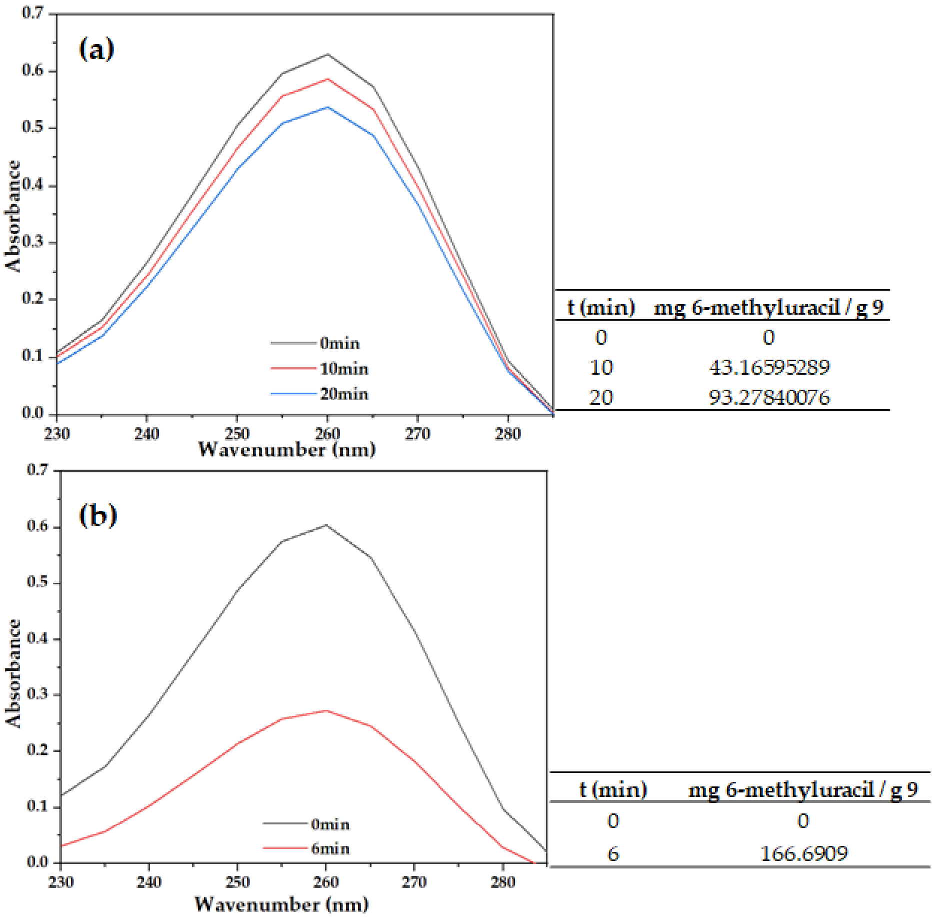

t (min)	mg 6-methyluracil / g 9
0	0
10	43.16595289
20	93.27840076

t (min)	mg 6-methyluracil / g 9
0	0
6	166.6909

Figure S10. UV studies of the 6-methyluracil adsorption by **9**; (**a**) 0.05mmol **9**/0.1 mmol 6-methyluracil and (**b**) 0.1 mmol **9**/0.1 mmol 6-methyluracil (in 10 mL of water).

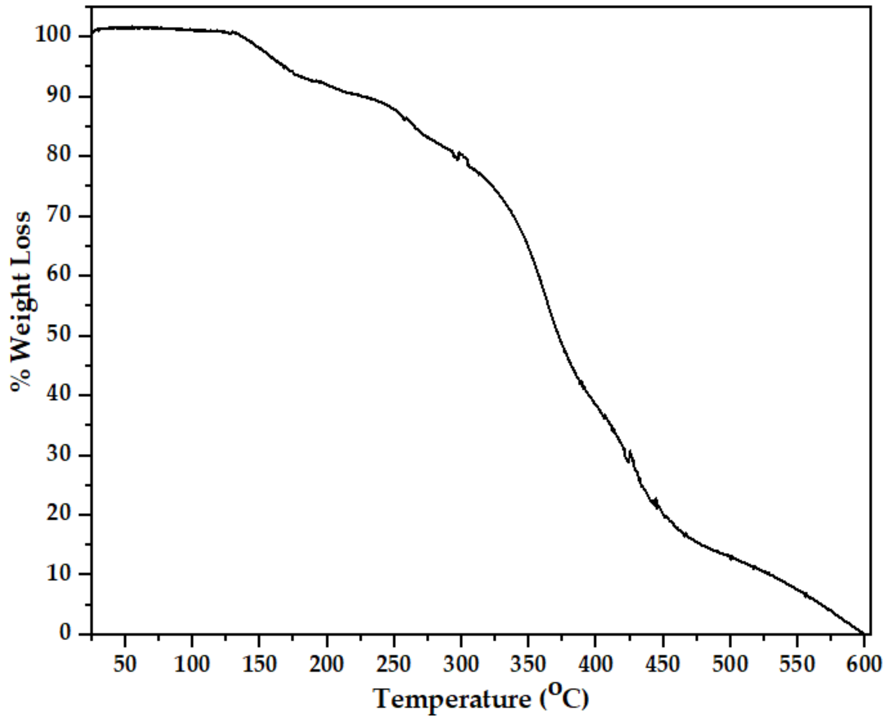

Figure S11. The TGA diagram for **1**.

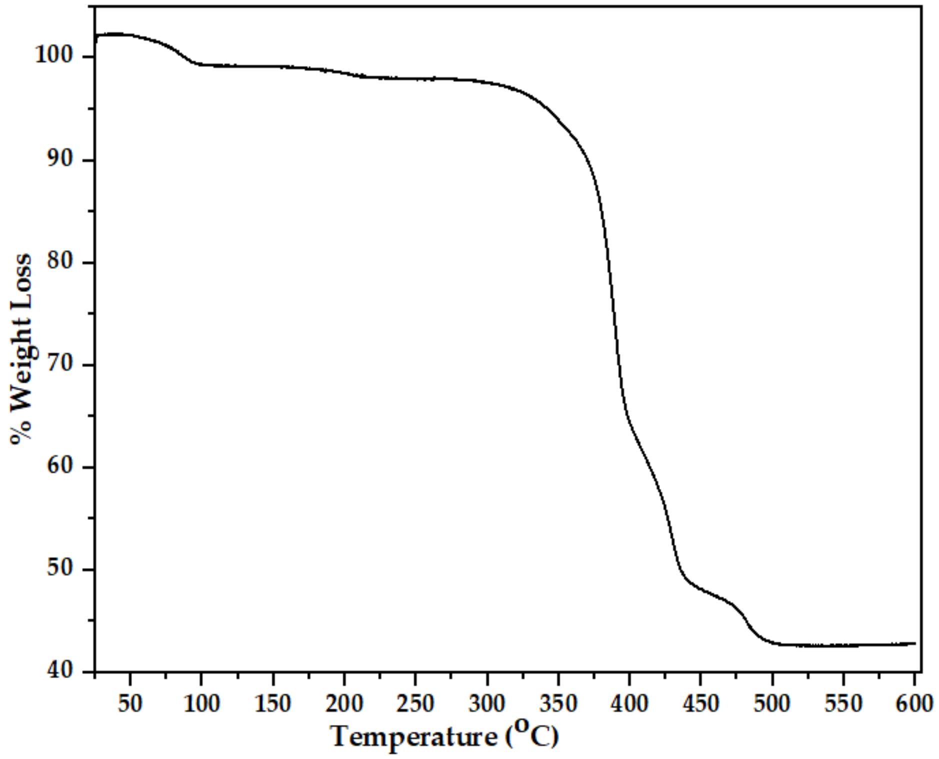

Figure S12. The TGA diagram for **2**.

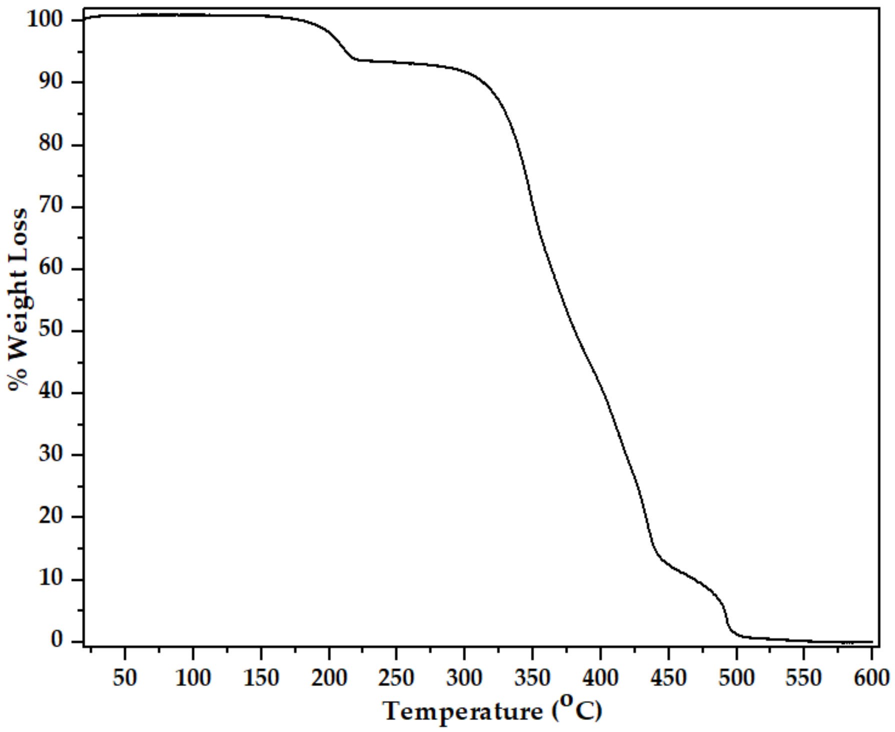

Figure S13. The TGA diagram for **3**.

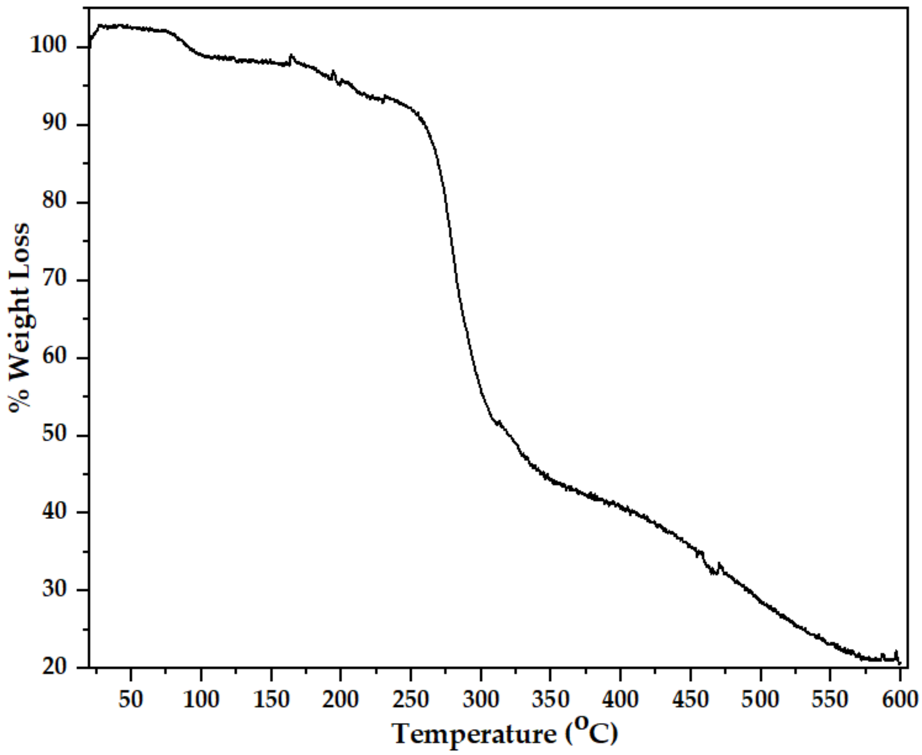

Figure S14. The TGA diagram for **6**.

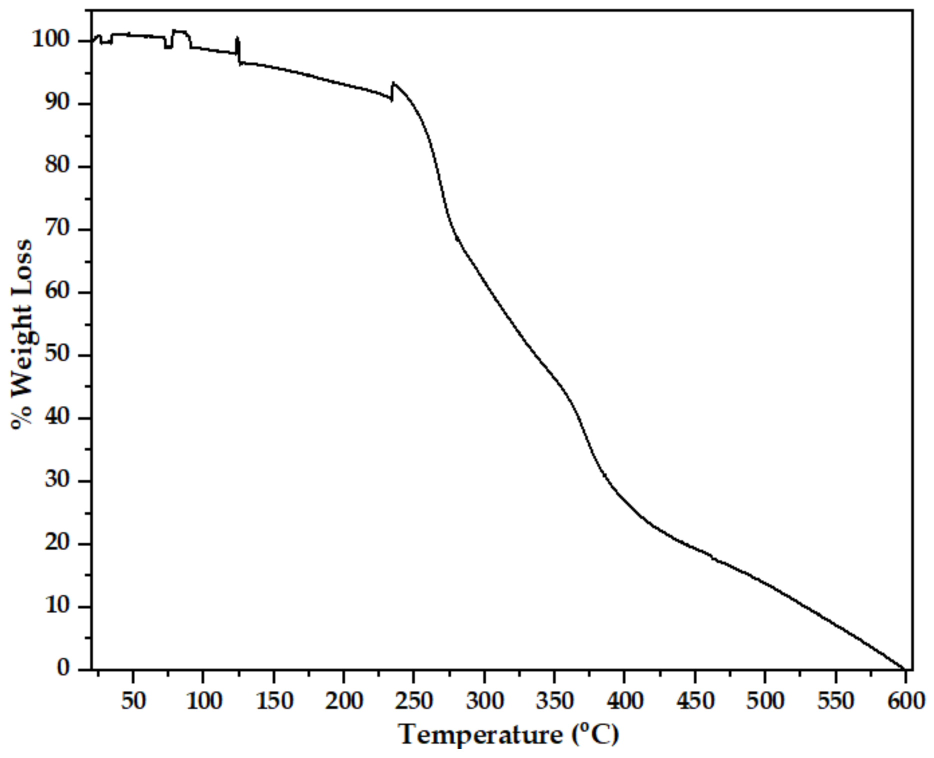

Figure s15. The TGA diagram for **7**.

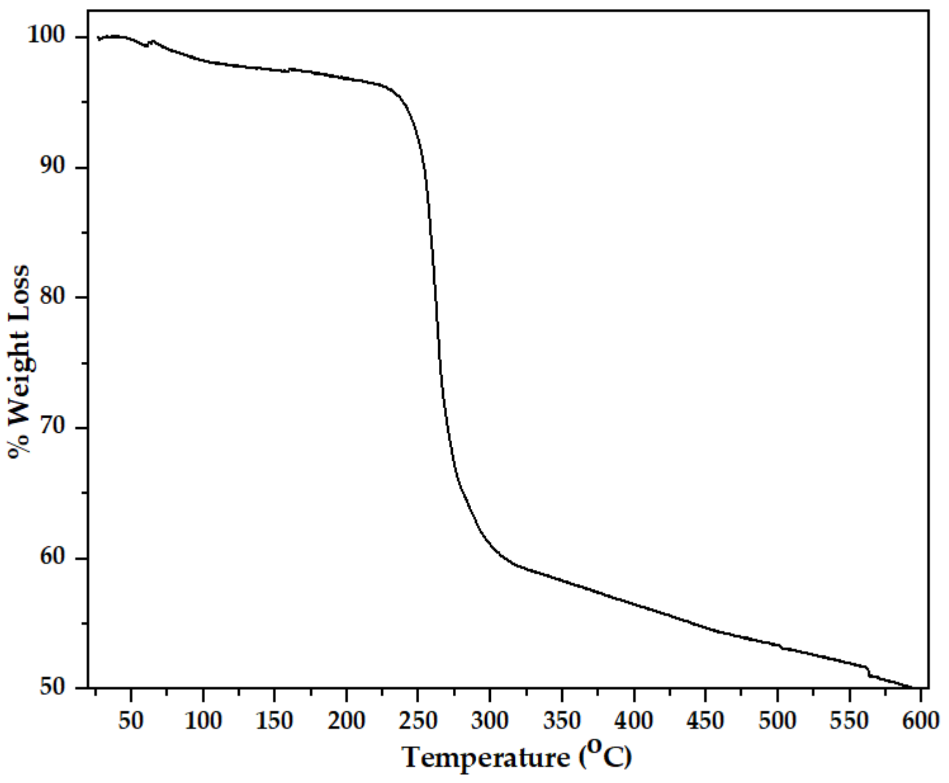

Figure S16. The TGA diagram for **8**.

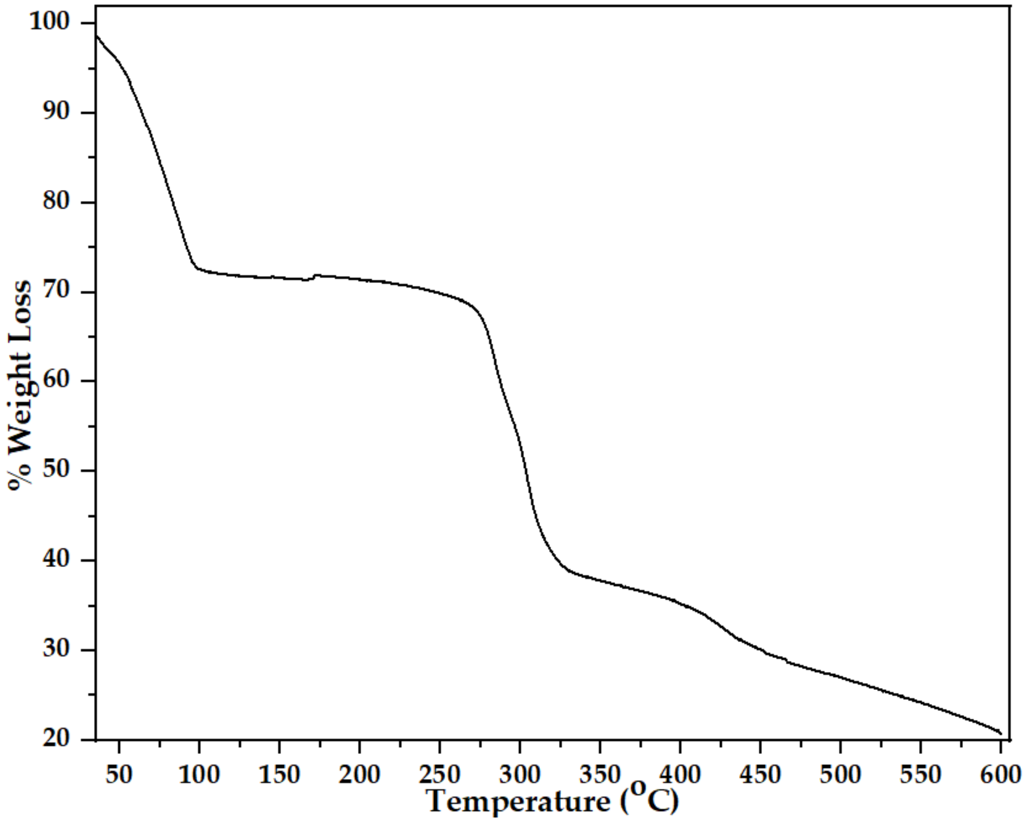

Figure S17. The TGA diagram for **9**.

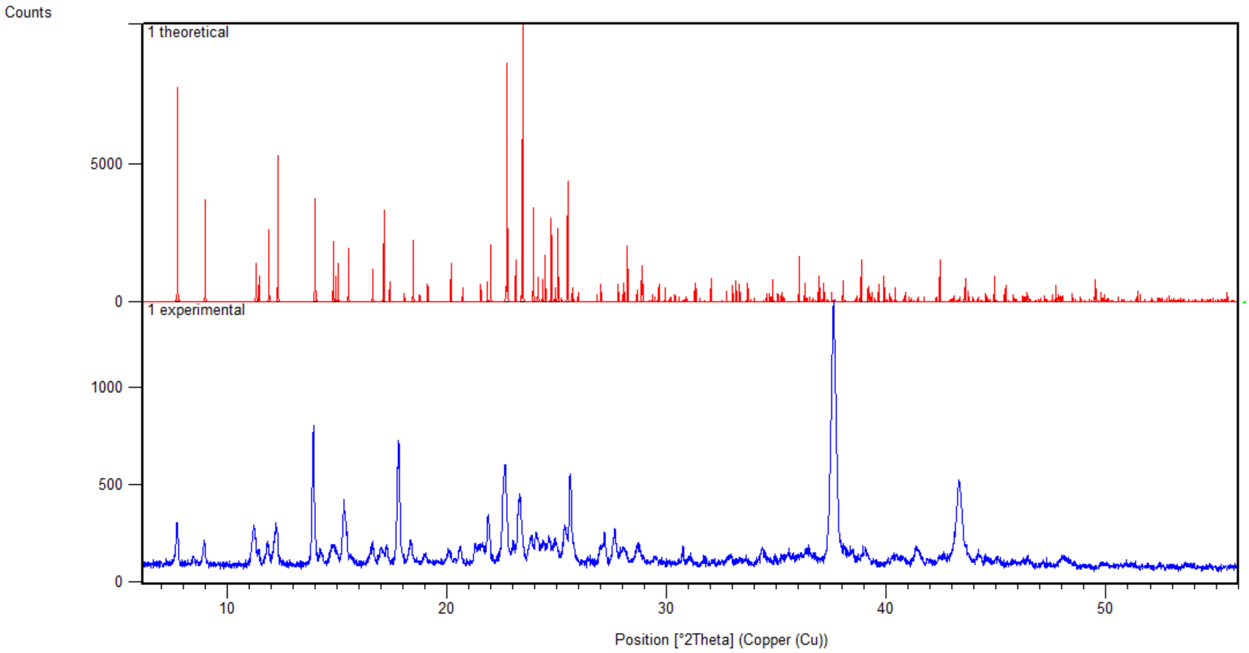

Figure S18. The experimental (bottom) and theoretical (top) pxrd pattern diagrams for **1**.

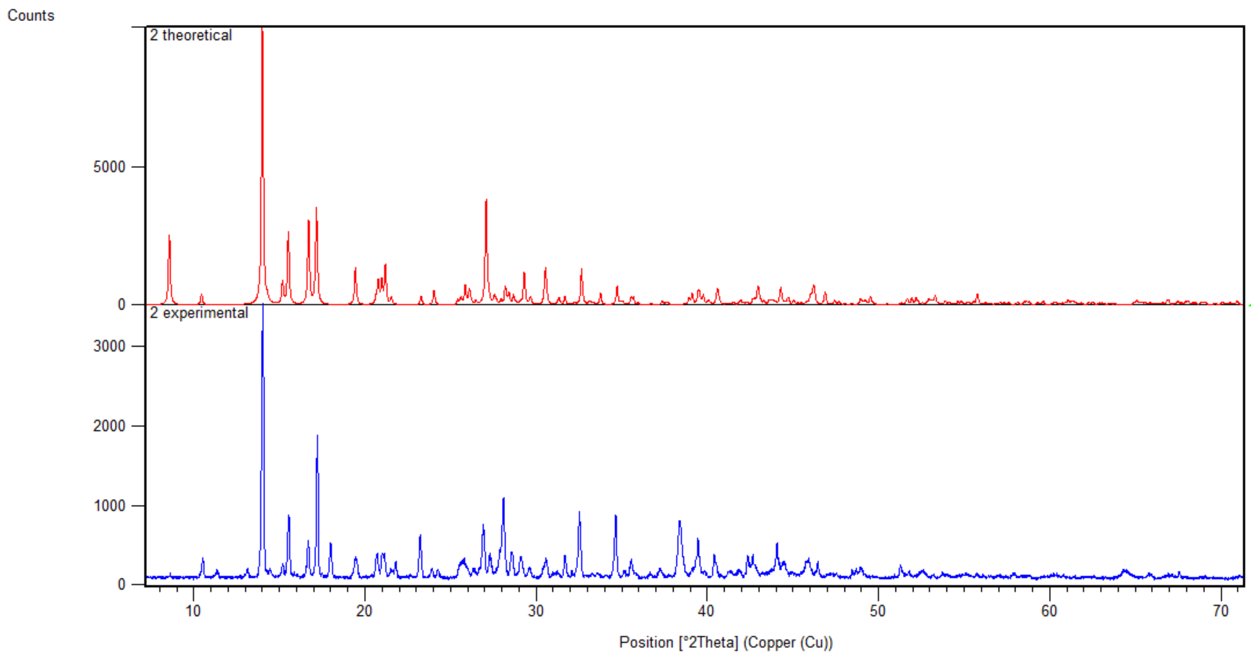

Figure S19. The experimental (bottom) and theoretical (top) pxrd pattern diagrams for **2**.

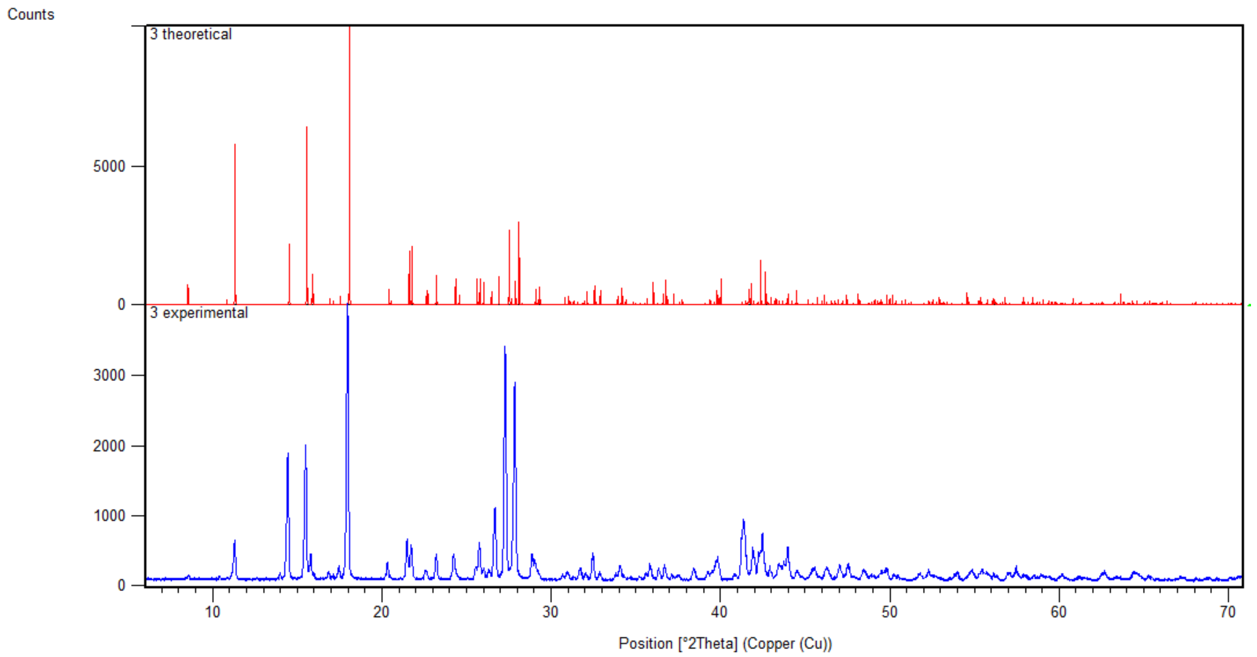

Figure S20. experimental (bottom) and theoretical (top) pxrd pattern diagrams for **3**.

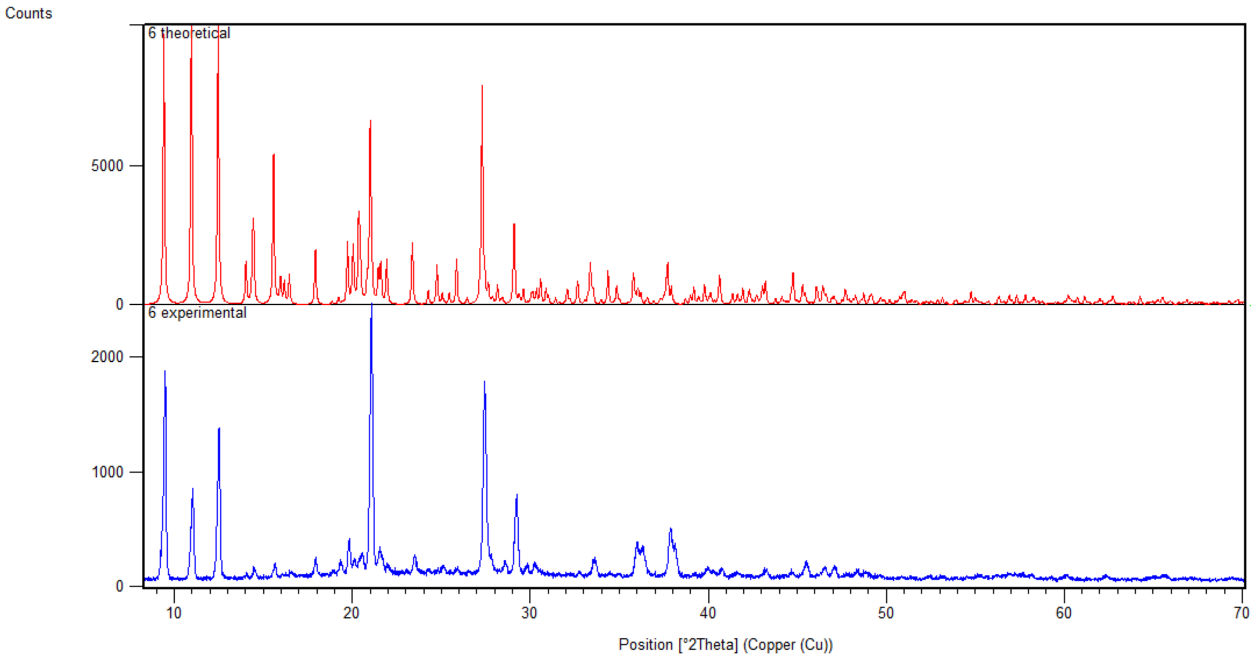

Figure S21. experimental (bottom) and theoretical (top) pxrd pattern diagrams for **6**.

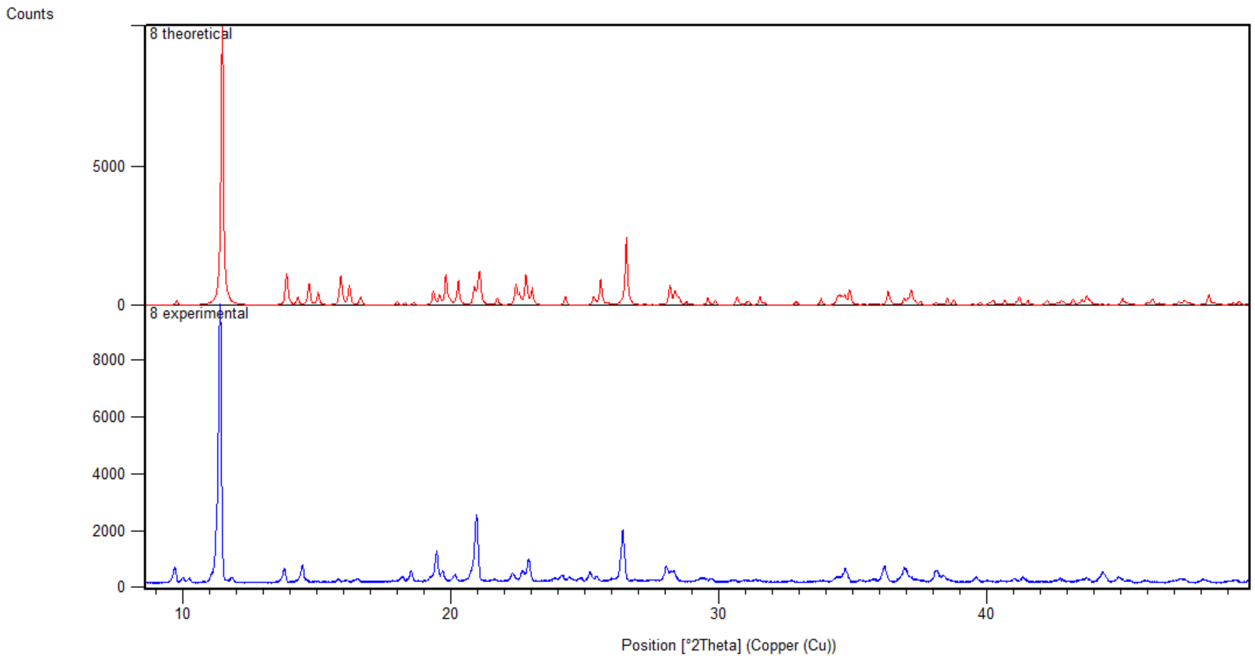

Figure S22. experimental (bottom) and theoretical (top) pxrd pattern diagrams for **8**.

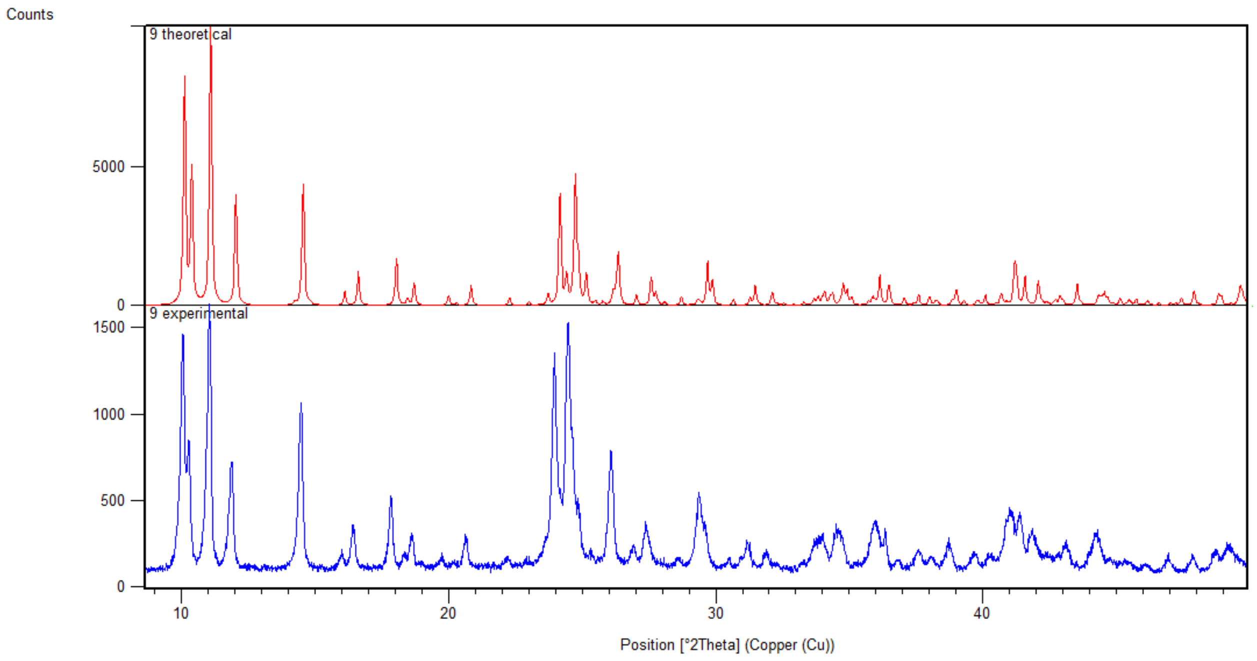

Figure S23. experimental (bottom) and theoretical (top) pxrd pattern diagrams for **9**.

© 2020 by the authors. Licensee MDPI, Basel, Switzerland. This article is an open access article distributed under the terms and conditions of the Creative Commons Attribution (CC BY) license (http://creativecommons.org/licenses/by/4.0/).
